# Voltage-Gated Sodium Channel, as an Ion Channel in Therapeutics: A Bibliometric Analysis of Global Trends (1984–2023)

**DOI:** 10.21315/mjms-01-2025-057

**Published:** 2025-06-30

**Authors:** Mohd Redhuan Mohd Noor, Siti Yusrina Nadihah Jamaludin, Mohd Harizal Senik, Muhammad Yusran Abdul Aziz

**Affiliations:** 1 Faculty of Medicine, Universiti Sultan Zainal Abidin, Medical Campus, Terengganu, Malaysia; 2 Faculty of Ocean Engineering Technology, Universiti Malaysia Terengganu, Terengganu, Malaysia; 3 Department of Neurosciences, School of Medical Sciences, Health Campus, Universiti Sains Malaysia, Kelantan, Malaysia; 4 UniSZA Science and Medicine Foundation Centre (PUSPA), Universiti Sultan Zainal Abidin, Gong Badak Campus, Terengganu, Malaysia

**Keywords:** VGSC, bibliometric analysis, ion channels, therapeutic, Scopus

## Abstract

Voltage-gated sodium channels (VGSCs) play a critical role in generating and propagating electrical signals in excitable cells. To date, the technology has gained much attention for its therapeutic potential in treating various diseases, including cancer. This study provides a comprehensive bibliometric analysis of VGSC research trends over 40 years (1984–2023) using the Scopus database. A total of 3,170 articles were analysed to explore publication productivity, authorship, key research topics, and collaboration patterns. The analysis identified the leading countries according to the number of articles contributed and determined that the USA, China, and Germany were the most productive. In fact, the USA alone accounts for 41.6% of global VGSC research on therapeutics. Additionally, based on the number of published articles, institutions such as Harvard Medical School and the University of Cambridge were found to be at the forefront of this field. Overall, the performed research focused on the therapeutic applications of VGSCs in conditions like atrial fibrillation, chronic pain, cancer, and genetic diseases. The study also highlights a growing interest in VGSC-related research in the past decade as a result of the advancements in molecular techniques such as next-generation sequencing (NGS) and small interfering RNA (siRNA). These findings suggest that VGSCs will remain a central point in therapeutic research for years to come, with a greater interest in international collaboration to drive further progress.

## Introduction

Voltage-gated sodium channels (VGSCs) are essential components in excitable cells, such as neurons and muscle cells, and are responsible for generating and propagating electrical signals. These channels are crucial in the molecular mechanisms involved in the development and transmission of action potentials. Over the past two decades, VGSCs have gained attention for their involvement in cancer metastasis, which positions them as potential therapeutic targets. Structurally, VGSCs comprise a single α-subunit (VGSCα) typically paired with auxiliary β-subunits (1). One of the distinctive features of VGSCs is their “loosely coupled knock-on” mechanism. This is a specialised function that allows sodium ions to permeate through the VGSC selectivity filter with partial coupling, where ion movements are influenced by the electrostatic effects of neighbouring ions rather than direct displacement. The mechanism is vital for ion permeability and selectivity (2).

VGSCs are organised into four homologous domains (I–IV), each containing six membrane-spanning helices (S1–S6) (3). Among these, the S4 segments serve as voltage sensors (4), while the S6 segments and the re-entrant loop located between S5 and S6 form the channel pore. In general, the α-subunit has a pseudo-symmetric structure, with the S5 and S6 segments from each domain arranged around the central pore to form the core configuration necessary for channel function (1). The S1–S3 segments provide structural stability and interaction points on the outer edge of the complex, while the S4 segment, centrally located within each domain, plays a critical role in voltage-dependent gating (5). VGSC α-subunits can also be further classified by their tetrodotoxin sensitivity and tissue distribution (6). Overall, this structural and functional diversity enables VGSCs to influence cancer progression, especially since some subtypes of VGSC, such as Na_V_1.5, can increase the local acidification around cancer cells by activating NHE-1, causing Na^+^/H^+^ exchange that acidifies perimembranous regions and promotes matrix degradation. Research by Sanchez-Sandoval et al. (7) and Horne et al. (8) has shown that VGSCs can alter intracellular pH and calcium levels, which directly impacts cell adhesion, migration, and invasion. Due to these critical characteristics, VGSCs are now considered promising yet underexplored therapeutic targets (7, 8).

Despite growing interest in VGSCs’ role in treating cancer, a specialised bibliometric study on VGSCs remains scarce. To date, only a limited number of studies have assessed trends and scientific contributions in this field from a global perspective. Although relatable bibliometric analyses on other ion channels provide valuable insights, such as Zhu et al.’s (9) work on atrial fibrillation and Xu et al.’s (10) study on the TRPV1 subtype, an in-depth exploration of VGSCs is required, given their emerging role in oncology. Previously, the Web of Science (WoS) database was often used for bibliometric research. However, for a better and more comprehensive analysis, the current research on VGSC chose Scopus as the main database to collect relevant information because of its extensive coverage and broader interdisciplinary.

This study aims to: i) analyse the temporal distribution of VGSC publications and therapeutic applications, ii) identify prominent authors, leading countries, and top academic institutions, iii) highlight common keywords and research themes, iv) determine countries with significant research contributions, and v) suggest future collaboration and research directions. It is expected that the findings from this study will equip researchers, policymakers, and stakeholders with an in-depth understanding of VGSC research trends, while emphasising critical areas for future investigation.

## Methods

A bibliometric study systematically analyzes global research trends within a specific topic or field by leveraging data from online databases. This method differs significantly from traditional review papers, as it uniquely examines the collective body of previous publications. Through this approach, researchers can identify the research direction, limitations, challenges, and achievements.

### Data Source and Search Strategy

The collection of relevant scientific articles on VGSCs from 1984–2026 (40 years) was conducted on 5 October 2024 using the Scopus database. The central theme of this study was ion channels, particularly VGSCs, and their therapeutic applications. In the early stage, the process began with text mining by inputting the search terms “ion* channel*” and “voltage AND gated AND sodium AND channel*,” followed by a series of terms representing therapeutic or treatment applications. Through the precise execution of text mining, using appropriate terms, data from textual documents, both linguistic and statistical, were clustered and transformed into visual representations for enhanced comprehension.

The initial search yielded 6,809 papers, with the earliest publication dating back to 1984 and the most recent in 2024. The specific query string used was TITLE-ABS (“ion* channel*”) AND (voltage AND gated AND sodium AND channel*) AND (“treatment*” OR “therapeutic*”). After refining the search to include only journal articles and conference proceedings from 1984 to 2023, the results were reduced to 3,491 documents. These results were categorised by year, source, author, affiliation, country/territory, subject area, and document type. Notably, this search string excluded standard review articles containing terms such as “progress,” “review,” “overview,” “updates,” “bibliometric,” and “potentiometric.”

**Figure 1:**
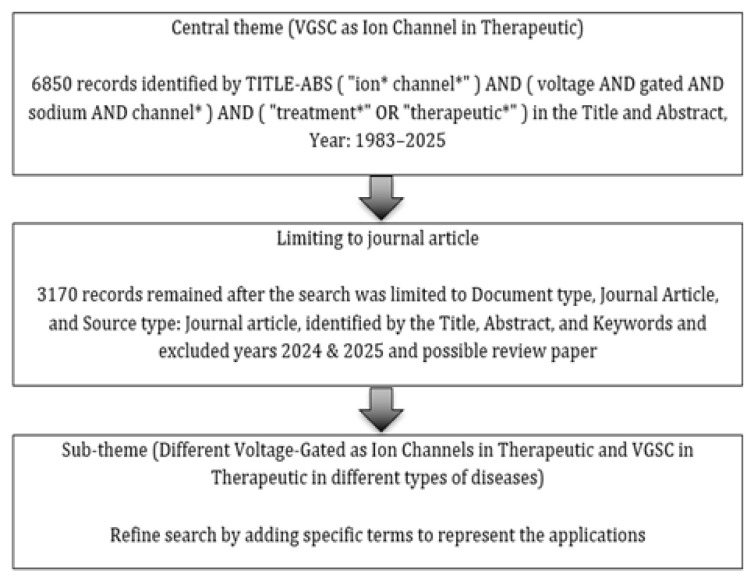
Research framework on the data collection process for primary and sub-themes

The AFFILCOUNTRY code was applied to extract single-country publications (SCP) data for specific countries to refine the search results. For the ranking assessments and data tabulation, bibliometric indicators available on Scopus were employed, including total publications, CiteScore, Field-Weighted Citation Impact (FWCI), total citations, and *h*-index. Additionally, two sub-themes were developed to explicitly examine the researcher’s interest in ion channel-related therapies across different diseases (Figure 1). These sub-themes categorised research by potassium, chloride, and calcium ion channels on cell membranes and VGSC applications in various diseases, such as infectious, deficiency, and physiological conditions. A separate search string was used for each ion channel. The sub-theme results were further examined for annual publication output among the top five contributing countries. Figure 1 presents the general data collection and refinement procedures, while Table S1 in the supplementary material details the search strings used for both main and sub-themes.

### Bibliometric Maps

Bibliometric maps, generated from 3,170 records in the Scopus database, were created using VOSviewer software version 1.6.18. This software was selected for its versatility and compatibility with major databases like Scopus, WoS, Dimensions, Lens, and PubMed. As a robust tool for visualising bibliometric data, VOSviewer can provide a precise representation of items of interest, such as countries and author keywords, and quantify the relationship strength between these items through numerical link values. Generally, it is understood that higher numerical link strengths reflect stronger connections. From this association, an intuitive interpretation of complex data can be made for an in-depth discussion of the findings.

The co-authorship analysis used link strength to represent the frequency of shared publications between countries. These publications were categorised based on co-authors’ affiliations, which represent joint publications and all of the involved countries or institutions. In comparison, the total link strength illustrates a country’s overall collaborative strength in relation to other countries. Similarly, in co-occurrence maps, link strength between author keywords signifies the frequency of their joint appearance across articles. Data for all tables and figures adhered to a full counting method, with detailed functionality available in the VOSviewer manual (11).

#### Co-authorship Assessment

A dataset involving 76 countries and 3,171 authors was included in the co-authorship analysis. These countries were categorised by continent: Asia, Europe, America, Africa, and Oceania. The resulting maps, presented in a network visualisation mode using a full counting system, underscore the global nature of the research field and the collaborative efforts of the scientific community.

#### Co-occurrence Assessment

Based on author keywords, the co-occurrence analysis involved 6,755 keywords extracted from the records. Before generating the co-occurrence maps, keywords were filtered by VOSviewer to reduce redundancy and ensure that each keyword has a distinct meaning. For example, terms like “ion channels,” “ion channeling,” and “ion channel medium” were consolidated under the keyword “ion channels.” This filtering process resulted in 6,715 unique keywords used for the maps. The minimum occurrence threshold for author keywords was set to the default value of 5, generating 290 keywords. The resulting maps were displayed in overlay visualisation mode using the full counting method, where the colour and link strength of items indicate the average publication year of documents containing the selected keywords.

### Multi-Ion Channels and VGSC Therapeutics in Various Disease Types

The central theme (keyword co-occurrences) and sub-themes (total number of publications) were compared to understand the broad scope of research on ion channels and their therapeutic applications. To achieve this, the search string was modified to include all publications that emphasised key terms related to ion channels and VGSC therapeutics across different disease types. Based on the collected records, leading countries were analysed according to their publication output related to the topics of interest.

## Results and Discussion

### Publication Productivity and Trends in Research Interest

**Figure 2:**
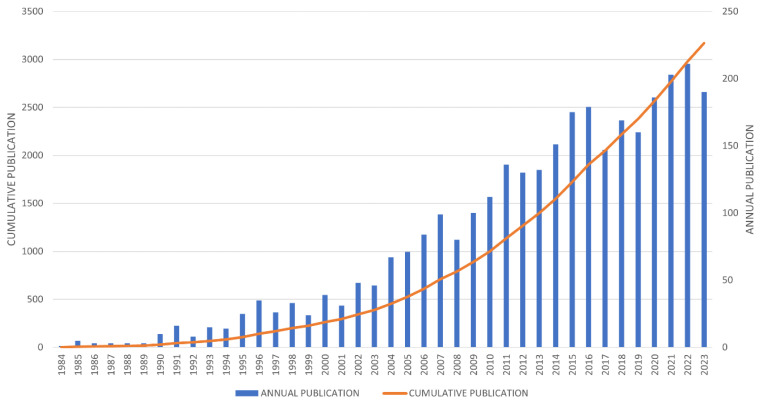
The annual cumulative publication and annual publication of a research article on VGSC as ion channel in therapeutic in Scopus 1984 to 2023

Over the past 40 years, from 1984 to 2023, a total of 3,170 articles have been published on VGSCs and their therapeutic applications. This substantial amount of work demonstrates significant growth and interest in this field (Figure 2). Prior to 1984, only one publication addressed sodium channels within therapeutic contexts. However, a steady rise in publication output then commenced and continued up to 1990, followed by a marked annual increase. Since 2009, annual publications have surpassed 100, with a significant surge from 2015, marking a pivotal period in VGSC research that indicates rapid advancements in therapeutic applications.

Many factors are considered responsible for this significant increase, including the widespread applications of VGSCs in medical research for cancer treatment, drug development, and other neurological conditions such as epilepsy and autism (4, 12–14). This trend is supported by Annual Growth Rate (AGR) metrics, which showed a remarkable 140% increase in 1993 compared to 1984 and a 470% rise in the number of published articles over the 40-year period.

Open access publications constitute 60% (1,902) of the 3,170 articles, underscoring the importance of accessible research in promoting global collaboration and faster knowledge dissemination. Many researchers, such as Dorta-González et al. (15) and Abd Wahid et al. (16), advocate for more work to be published in open access to enhance citations and awareness of VGSC-related studies worldwide.

Research on VGSCs covers multiple disciplines, with prominent contributions in biochemistry, genetics, and molecular biology (1,617 articles), followed by medicine (918 articles), neuroscience (762 articles), and pharmacology, toxicology, and pharmaceutics (678 articles). In particular, the forefront coverage of biochemistry, genetics, and molecular biology was due to the technological advancements of molecular sequencing techniques like small interfering RNA (siRNA), which have significantly driven VGSC research and facilitated the discovery of ion channel blockers (14, 17–20). In regards to the languages, most publications were published in English (3,114 articles, or 98.2%), with a minority in Chinese (15; 0.5%), German (12; 0.4%), French (11; 0.4%), and other languages (18; 0.5%).

### Preferred Journals

An analysis of the top 10 most productive journals in VGSC therapeutic research revealed Elsevier and John Wiley and Sons as the most prominent publishers, each represented by two journals. The Elsevier was represented through *Brain Research* and *Neuroscience*, whereas the John Wiley and Sons was represented through the *Journal of Physiology* and the *British Journal of Pharmacology* (Table 1). The remaining journals were distributed across different publishers. *PLOS ONE* was ranked as the most prolific journal with 84 published articles, which accounted for 2.64% of all VGSC research articles. This was followed by the *Journal of Biological Chemistry* with 68 articles (2.15%) and the *Journal of Neuroscience* and *Proceedings of the National Academy of Sciences of the United States of America*, each with 66 articles (2.08%). Although *PLOS ONE* has the highest number of publications, the *Journal of Biological Chemistry* demonstrated a higher citation impact, suggesting that citation metrics might better reflect a journal’s influence than sheer publication volume. An evaluation based on CiteScore revealed that *Proceedings of the National Academy of Sciences of the United States of America* achieved the highest CiteScore of 19 despite ranking fourth in publication count. The *Journal of Neurophysiology*, however, presented the lowest CiteScore at 4.8, with nine of the top 10 journals achieving a CiteScore above 5. In general, CiteScore is considered a vital indicator of a journal’s reach and influence. As such, high CiteScore values often attract more audiences to the journals for their publication needs.

Apart from CiteScore, FWCI also serves as an essential metric. This parameter reflects the ratio of total citations an article receives compared to the expected number for its field. For instance, an FWCI of 1 signifies citations at an average rate globally, while values above 1 indicate higher-than-expected citation rates for the article with respect to other articles of similar topics. Notably, the most cited article from the *British Journal of Pharmacology* achieved the highest FWCI of 28.64. After a thorough assessment, it is postulated that this paper received much attention because it successfully organises the presentation of target drugs and clarifies consistency in pharmacology fields referring to the International Union of Basic and Clinical Pharmacology (IUPHAR) and the British Pharmacological Society (BPS) guidelines. To conclude, the authors should consider both CiteScore and FWCI in journal selection and review the journal’s past publications to ensure alignment with their target readership.

### Leading Countries and Institutions

Table 2 highlights the top 15 countries in the global VGSC research field. Overall, the USA, China, and Germany collectively contributed approximately 66% of the worldwide publication output. As the main contributor, the USA accounted for 1,318 publications, representing 41.6% of global VGSC research, followed by China and Germany, each producing approximately half as many publications as the USA. Harvard Medical School emerged as the most productive institution in the USA and ranked highest in total publications (TPi) among institutions within these top 15 countries. In contrast, KU Leuven from Belgium ranked the lowest, with only two publications managed to be produced at the institutional level. The analysis of SCP revealed varying collaboration patterns. Notably, China (69.2%), Japan (69.7%), and South Korea (71.83%) exhibited high SCP rates, suggesting robust intra-country collaboration with limited international co-authorship. Despite leading in total publications, the USA’s SCP rate stood at 64.64%, indicating a relatively higher level of intra-country collaboration. Conversely, Belgium demonstrated the lowest SCP rate at 5.8%, with 65 out of 69 publications involving authors from 76 countries. This high international collaboration rate highlights Belgium’s extensive network and openness to global partnerships, characteristics that promote a rich exchange of knowledge and expertise. South Korea’s SCP dominance reflects a strong national focus, with Seoul National University College of Medicine producing 71.83% of its VGSC research without international affiliations. Among top institutions, Harvard Medical School, the University of Cambridge, and the University of Toronto were highly ranked in the QS World University Rankings 2025. This illustrates increasing engagement in VGSC therapeutic research among the most advanced institutions in the world, underscoring the field’s growing appeal within the scientific community (21).

**Figure 3:**
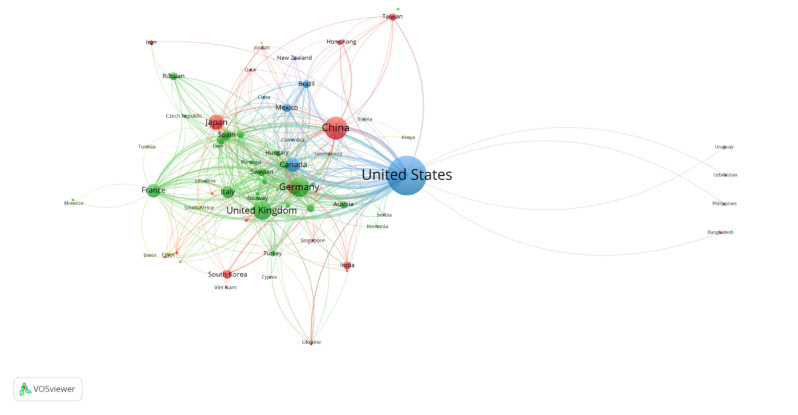
A screenshot of a bibliometric map created based on co-authorships with network visualisation mode (https://tinyurl.com/293o7qye)

Figure 3 depicts the global distribution of VGSC research, with Europe leading in the number of contributing countries (34), followed by Asia (23), America (11), Africa (7), and Oceania (1). In co-authorship analysis, the USA maintained the highest number of affiliations, collaborating with 59 countries across 659 co-authorship instances. The United Kingdom ranked second, with links to 49 countries and 314 co-authorships, while Italy, France, and Canada also showed substantial international collaboration, with 39, 38, and 36 links, respectively. Over half of the 76 listed countries exhibited low collaborative activity, with fewer than ten collaborative publications. Algeria, Bulgaria, Iceland, Morocco, the Philippines, Uruguay, and Venezuela each contributed only one collaborative publication. It is suggested that factors such as international networks, visiting scholars, and foreign postgraduate students affect the collaborative intensity in certain countries. The situation emphasises the importance of global partnerships in fostering impactful and wide-reaching VGSC research. Therefore, by increasing international collaboration, resilient and globally beneficial research outputs can be cultivated for a better future.

### Leading Authors

Table 3 presents the top 10 most prolific authors in VGSC therapeutic research. To sum up, these researchers represent four countries: Australia (four authors), the USA (three authors), Belgium (two authors), and China (one author). Glenn F. King and Guirong Li were noted as early pioneers in ion channel research for therapeutic applications, with their initial publications dating back to 1983. Glenn’s first study, “Proton NMR spectroscopic studies of dipeptidase in human erythrocytes,” received 21 citations, while his most cited paper in 2021, “Trends in peptide drug discovery,” received 973 citations, illustrating his evolving focus on ion channels. Guirong Li’s early work in 1983, “Effects of dauricine on the dose-effect response of isoprenaline and calcium and the electro-mechanic activity of cat papillary muscle,” garnered two citations initially but reached 724 citations for his influential paper in 1997, “Ionic remodeling underlying action potential changes in a canine model of atrial fibrillation.” Both authors have consistently explored ion channels as therapeutic targets since the 1980s. Among the authors listed, publication dates ranged from 1981 to 2008, with six out of ten authors acting as the lead authors in their initial studies.

Although the ranking was based on data from Scopus, other than the number of publications, there were no formal rules for author sequence determination. As such, readers can interpret rankings based on other metrics such as total citations or *h*-index. By definition, the *h*-index is a metric that measures productivity and citation impact. In sum, Michael John Ackerman, ranked seventh, has the highest *h*-index of 135, with a total of 1,610 citations. However, in terms of author publication count, Jan Tytgat from Belgium led the list with 37 papers since 1988, with an *h*-index of 52 and 515 citations. Steve Peigneur from Belgium and Glenn F. King from Australia ranked second and third, respectively, each accumulating over 400 citations. Interestingly, Peigneur, who began publishing in 2008, rose quickly to a top position despite his late entry. The phenomenon reflects the field’s rapid growth and substantial attention from researchers worldwide.

When cross-checking the affiliations, it was found that Peigneur and Tytgat were both recently affiliated with KU Leuven’s Department of Pharmaceutical and Pharmacological Sciences. Similarly, four of the top ten authors, Glenn F. King, Richard James Lewis, Irina Vetter, and Paul F. Alewood, were from the University of Queensland. Both institutions, KU Leuven in Belgium and the University of Queensland in Australia, were previously identified as the most influential in the field. This discovery underscores the role of the institutions as the centre for VGSC studies.

### Author Keywords

VOSviewer identified 6,693 unique author keywords from the collected database. To enhance consistency and reduce redundancy, keywords with similar or synonymous meanings were consolidated to avoid redundancy. Following refinement, a total of 270 keywords met the threshold for inclusion when the minimum occurrence was set at five.

#### Terminology and Concepts

**Figure 4:**
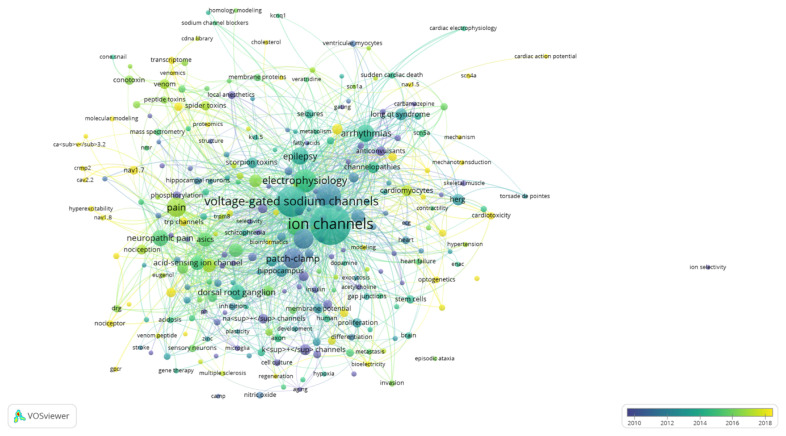
Research trends of the selected significant application in ion channel as a treatment on overlay visualisation (https://tinyurl.com/2y66ml7l)

An assessment of the results in Figure 4 gave an unexpected result: “ion channels” had the highest occurrence, with 359 instances and 188 links to other keywords. The most frequently occurring terms included “voltage-gated sodium channels” (203 occurrences, 155 links), followed by “voltage-gated potassium channels” (133 occurrences, 101 links), and “electrophysiology” (114 occurrences, 107 links). The frequent selection of “ion channel” as an author keyword is likely due to its relevance as a primary topic within voltage-gated ion channels in membrane cells. “Ion channels” represent the primary classification, with VGSCs being one of the sub-classifications.

The relationship between “ion channels” and other keywords was significant, with a total link strength of 569. Specifically, “ion channels” showed a link strength of 19 with “voltage-gated sodium channels,” 15 with “voltage-gated potassium channel,” six with “calcium channels,” and two with “chloride channels.” The terminology “ion channels” and “voltage-gated sodium channels” showed the most relationship in this trend.

#### Topic of Interest

The cumulative and annual publication trends on VGSCs in therapeutic applications from 1984 to 2023 demonstrated a steady increment, underscoring increasing interest in this field. Advancements in technology have facilitated the identification of VGSC molecular subtypes and expanded their potential therapeutic applications. Prominent keywords in VGSC subtype research, as shown in Figure 4, include “Na_V_ 1.5” (occurrence: 7, average publication year: 2020), “neonatal Na_V_ 1.5” (occurrence: 13, average publication year: 2020), “Na_V_ 1.7” (occurrence: 17, average publication year: 2018), and “Na_V_ 1.8” (occurrence: 7, average publication year: 2020), with studies often focusing on their roles in pain modulation and cardiovascular conditions such as atrial fibrillation, arrhythmia, and Brugada syndrome. Subtypes of VGSCs promise a new potential therapeutic view since many research studies have shown a significant relationship between subtypes of VGSCs and diseases. Hence, up until the period of data collection, research on all subtypes was still trending, with the average number of articles published in 2020.

Research on specific VGSC subtypes, such as neonatal Na_V_ 1.5, has expanded into oncology, including studies on breast cancer (22, 23), brain cancer (24), colorectal cancer (19), lung cancer (25), and ovarian cancer (17). VOSviewer analysis in Figure 4 highlights that most VGSC research within therapeutic applications has concentrated in the past decade, signalling a recent surge in interest. Areas such as heart failure (occurrence: 9, average publication year: 2015), hypertension (occurrence: 6, average publication year: 2016), cancer (occurrence: 16, average publication year: 2018), and chronic pain (occurrence: 9, average publication year: 2018) reflect this trend, suggesting significant potential for future research into VGSCs across diverse therapeutic contexts.

### Distribution of Publications on Different Ion Channels and VGSC Therapeutics in Other Disease Types

**Figure 5:**
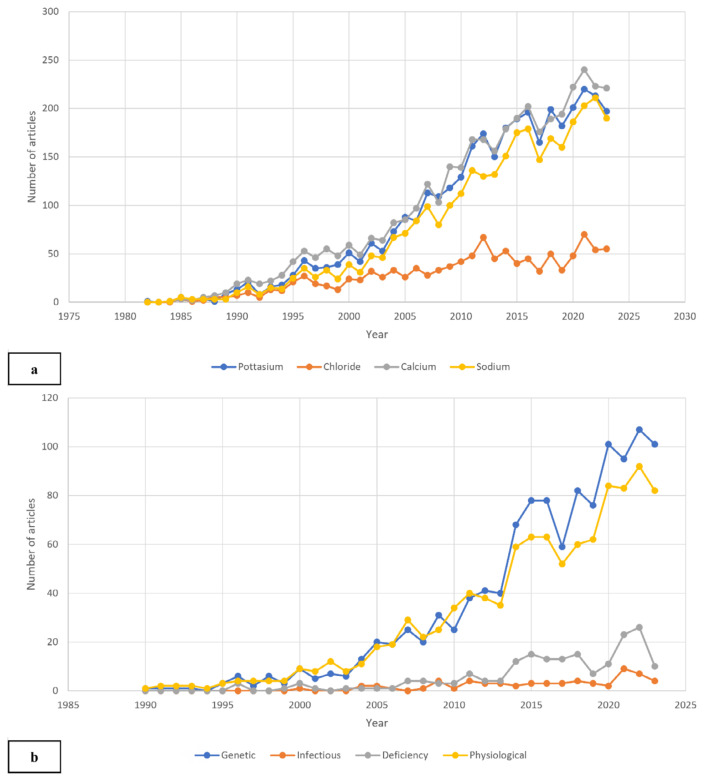
Research trends on the (a) different ion channels and (b) VGSC as a treatment for different types of disease

Figure 5(a) illustrates publication trends across various ion channels, including VGSCs, calcium, potassium, and chloride channels. While publications on chloride channels have steadily declined since 2000, the number of studies on calcium (3,917 publications), potassium (3,628 publications), and sodium channels (3,170 publications) has more than doubled within five years after 2000. The bibliometric analysis presented in Figure 4 further indicates that VGSC therapeutics were more prominent than other ion channels, with VGSC-related studies having an average publication year of 2013, in contrast to 2011 for potassium and calcium channels and 2007 for chloride channels.

Figure 5(b) categorises VGSC applications across various diseases, including infectious, deficiency, genetic, and physiological diseases. Notably, VGSCs were most frequently studied in relation to genetic diseases, with 1,168 publications compared to 1,032 for physiological diseases, 186 for deficiency diseases, and 62 for infectious diseases. Over the last five years (2019–2023), nearly 480 papers, representing 40% of all VGSC-related studies, focused on genetic disease therapies. This surge reflects advancements in molecular techniques, particularly next-generation sequencing (NGS), which have facilitated the identification of potential blockers to prevent cancer metastasis, as introduced by Guan et al. (26). Additionally, Gibbs et al. (27) demonstrated that NGS could enhance cancer survival outcomes by evaluating progression-free survival and overall survival among cancer patients. Collectively, these recent VGSC therapeutic and genetic disease approaches signal promising future research directions.

**Figure 6:**
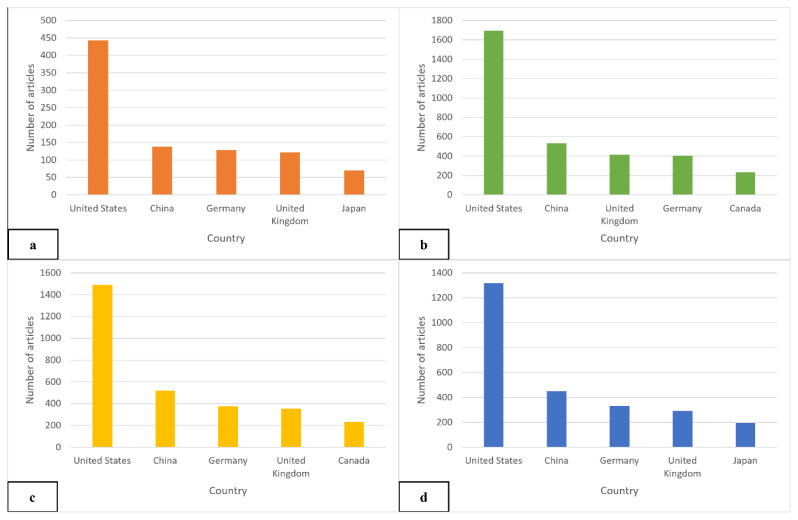
Five countries with the most publications on the different ion channels in therapeutic (a) chloride channels, (b) calcium channels, (c) potassium channels, and (d) sodium channels

**Figure 7:**
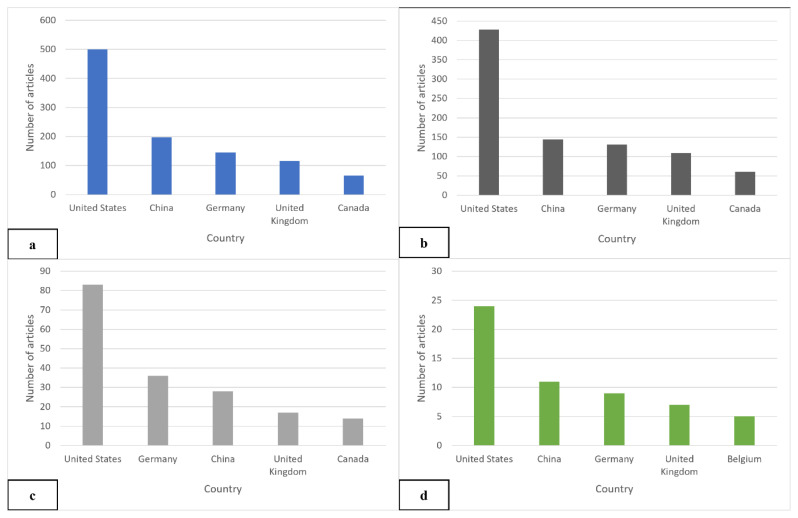
Five countries with the most publications on the VGSC therapeutic in different types (a) genetic disease, (b) physiological disease, (c) deficiency disease, and (d) infectious disease

Furthermore, Figures 6 and 7 illustrate the top five countries contributing most significantly to VGSC and ion channel research. As expected, both the USA and China emerged as leaders in this domain. These countries, ranked as the top two globally by gross domestic product (GDP) in 2024, reflect the ongoing prospects for growth in this field. Although Malaysia has published only four papers, with an average publication year of 2018, the country demonstrates immense potential for future growth with the appropriate support.

### Limitations of the Study

Some author keywords may have been excluded from the co-occurrence analysis in VOSviewer due to incomplete information in the journals. Therefore, future studies should consider incorporating additional databases, such as the WoS, to achieve a more comprehensive and detailed bibliometric analysis.

## Conclusion

This study provides a comprehensive analysis of VGSCs research trends in therapeutic applications over the past 40 years (1984–2023). Drawing on data from the Scopus database, a total of 3,170 journal articles and conference proceedings were retrieved and examined. In addition, stringent filtering criteria were applied before data collection to omit unrelated works and review articles. Although publication growth was initially insignificant, it has more than doubled since 2005. Based on this trajectory, VGSC research is expected to continue expanding for years to come. Notably, institutions in the top 100 World University Rankings, such as Harvard and Cambridge, were increasingly involved in this field. Their extensive involvement enhances the field’s visibility and credibility. Interestingly, despite Belgium’s low single-country publication rate, researchers from KU Leuven ranked among the top contributors, highlighting the significant potential for international collaborations. In conclusion, while sodium channels remain a central focus of therapeutic research, the increasing interest in potassium and calcium channels reflects a broadening scope in ion channel studies, suggesting promising directions for future exploration.

## Supplementary Material

**Table S1:** The search strings used in this study

Item	Theme	Topic of interest	Search string
1.	Central	Voltage-Gated Sodium channel in therapeutic research articles (6,850 records)	TITLE-ABS ( “ion* channel*” ) AND ( voltage AND gated AND sodium AND channel* ) AND ( “treatment*” OR “therapeutic*” )
2.	Central	Exclude the years 2025 and 2024. Limit to Journal article (3,491 records)	TITLE-ABS ( “ion* channel*” ) AND ( voltage AND gated AND sodium AND channel* ) AND ( “treatment*” OR “therapeutic*” ) AND ( EXCLUDE ( PUBYEAR, 2024 ) OR EXCLUDE ( PUBYEAR, 2025 ) ) AND ( LIMIT-TO ( DOCTYPE, “ar” ) ) AND ( LIMIT-TO ( PUBSTAGE, “final” ) ) AND ( LIMIT-TO ( SRCTYPE, “j” ) )
3.	Central	Possible review article (321 records)	TITLE-ABS ( “ion* channel*” ) AND ( voltage AND gated AND sodium AND channel* ) AND ( “treatment*” OR “therapeutic*” ) AND ( EXCLUDE ( PUBYEAR, 2024 ) OR EXCLUDE ( PUBYEAR, 2025 ) ) AND ( LIMIT-TO ( DOCTYPE, “ar” ) ) AND ( LIMIT-TO ( PUBSTAGE, “final” ) ) AND ( LIMIT-TO ( SRCTYPE, “j” ) ) AND (TITLE (recent OR progress OR review OR critical OR revisit OR advance* OR highlight OR perspective OR prospect OR trends OR bibliometric OR scientometric OR insights OR overview OR “state of the art” OR challenges OR updates) OR ABS (progress OR review OR bibliometric OR scientometric ) )
4.	Central	Final (3,170 records)	TITLE-ABS ( “ion* channel*” ) AND ( voltage AND gated AND sodium AND channel* ) AND ( “treatment*” OR “therapeutic*” ) AND NOT EID ( 2-s2.0-84927597176 OR 2-s2.0-84922010899 OR 2-s2.0-84900395214 OR 2-s2.0-85107710767 OR 2-s2.0-84962866792 OR 2-s2.0-33846673828 OR 2-s2.0-33947512419 OR 2-s2.0-85020991556 OR 2-s2.0-85013188047 OR 2-s2.0-84867125510 OR 2-s2.0-84891480845 OR 2-s2.0-84879845514 OR 2-s2.0-84942895036 OR 2-s2.0-84897059401 OR 2-s2.0-30844471383 OR 2-s2.0-85007352588 OR 2-s2.0-85039985438 OR 2-s2.0-0042536473 OR 2-s2.0-85021167561 OR 2-s2.0-79960698168 OR 2-s2.0-0037762690 OR 2-s2.0-85007368482 OR 2-s2.0-79955768829 OR 2-s2.0-84936928499 OR 2-s2.0-84951574682 OR 2-s2.0-70349335964 OR 2-s2.0-34447098802 OR 2-s2.0-85059541431 OR 2-s2.0-0030729279 OR 2-s2.0-77952309581 OR 2-s2.0-84883246797 OR 2-s2.0-85056496309 OR 2-s2.0-0025318574 OR 2-s2.0-85065493621 OR 2-s2.0-85060133773 OR 2-s2.0-0035313235 OR 2-s2.0-84962920731 OR 2-s2.0-84916207732 OR 2-s2.0-85046630803 OR 2-s2.0-84913549379 OR 2-s2.0-85007289400 OR 2-s2.0-36749015585 OR 2-s2.0-85066149701 OR 2-s2.0-79955558300 OR 2-s2.0-85073657423 OR 2-s2.0-85037690174 OR 2-s2.0-85074871955 OR 2-s2.0-84858161220 OR 2-s2.0-0023892933 OR 2-s2.0-79952262290 OR 2-s2.0-84981489482 OR 2-s2.0-84916201722 OR 2-s2.0-84890879754 OR 2-s2.0-84878393994 OR 2-s2.0-0037220090 OR 2-s2.0-84899860722 OR 2-s2.0-0036062999 OR 2-s2.0-84875794865 OR 2-s2.0-84958582085 OR 2-s2.0-84958242104 OR 2-s2.0-33750529349 OR 2-s2.0-85044159990 OR 2-s2.0-80054860325 OR 2-s2.0-84963751071 OR 2-s2.0-84893288265 OR 2-s2.0-85058928631 OR 2-s2.0-0031014473 OR 2-s2.0-84882705859 OR 2-s2.0-84880597393 OR 2-s2.0-84940413152 OR 2-s2.0-84877986414 OR 2-s2.0-0031030995 OR 2-s2.0-84883592437 OR 2-s2.0-33846264865 OR 2-s2.0-0030013925 OR 2-s2.0-84863777217 OR 2-s2.0-79959542489 OR 2-s2.0-58349114016 OR 2-s2.0-33745066809 OR 2-s2.0-84868126752 OR 2-s2.0-84866179386 OR 2-s2.0-84923819161 OR 2-s2.0-0032473367 OR 2-s2.0-70350379630 OR 2-s2.0-84925068443 OR 2-s2.0-33846188141 OR 2-s2.0-85078793098 OR 2-s2.0-84867899587 OR 2-s2.0-0036711450 OR 2-s2.0-84942886554 OR 2-s2.0-84921762521 OR 2-s2.0-84907515649 OR 2-s2.0-84864851318 OR 2-s2.0-0033066629 OR 2-s2.0-85082191409 OR 2-s2.0-84953297333 OR 2-s2.0-84945961579 OR 2-s2.0-84983788995 OR 2-s2.0-84871195861 OR 2-s2.0-85029213913 OR 2-s2.0-79955781475 OR 2-s2.0-84994959932 OR 2-s2.0-0033796095 OR 2-s2.0-84954285851 OR 2-s2.0-84896257270 OR 2-s2.0-85020011420 OR 2-s2.0-85058417690 OR 2-s2.0-85026610521 OR 2-s2.0-35648965716 OR 2-s2.0-84898772762 OR 2-s2.0-0034765967 OR 2-s2.0-85039868873 OR 2-s2.0-85083345183 OR 2-s2.0-84937808968 OR 2-s2.0-85071668624 OR 2-s2.0-85053042853 OR 2-s2.0-85007502838 OR 2-s2.0-85071777987 OR 2-s2.0-80052018704 OR 2-s2.0-84975781011 OR 2-s2.0-0027970905 OR 2-s2.0-85008474476 OR 2-s2.0-84994460502 OR 2-s2.0-84987851765 OR 2-s2.0-85070461451 OR 2-s2.0-79952082416 OR 2-s2.0-34547928925 OR 2-s2.0-85023744316 OR 2-s2.0-84889071254 OR 2-s2.0-77956830773 OR 2-s2.0-85112324505 OR 2-s2.0-84866179225 OR 2-s2.0-84922469457 OR 2-s2.0-0033016358 OR 2-s2.0-85118244016 OR 2-s2.0-85066492387 OR 2-s2.0-84869435941 OR 2-s2.0-85008702040 OR 2-s2.0-85041405270 OR 2-s2.0-85009442806 OR 2-s2.0-85065876846 OR 2-s2.0-84862023506 OR 2-s2.0-84930200978 OR 2-s2.0-84925340657 OR 2-s2.0-84895197157 OR 2-s2.0-84909583123 OR 2-s2.0-84931275456 OR 2-s2.0-85065229902 OR 2-s2.0-84911422091 OR 2-s2.0-79251630714 OR 2-s2.0-85097099229 OR 2-s2.0-84930644403 OR 2-s2.0-85131739568 OR 2-s2.0-84944047911 OR 2-s2.0-84952914192 OR 2-s2.0-84947802952 OR 2-s2.0-85111296029 OR 2-s2.0-84903791068 OR 2-s2.0-84942112073 OR 2-s2.0-0032886030 OR 2-s2.0-84930628571 OR 2-s2.0-84961684980 OR 2-s2.0-85043599838 OR 2-s2.0-84961218926 OR 2-s2.0-85130511821 OR 2-s2.0-0023296279 OR 2-s2.0-85093918416 OR 2-s2.0-50649102821 OR 2-s2.0-84867481677 OR 2-s2.0-84873371685 OR 2-s2.0-84866927563 OR 2-s2.0-34249790552 OR 2-s2.0-77953806557 OR 2-s2.0-84883057159 OR 2-s2.0-85018824585 OR 2-s2.0-85018255401 OR 2-s2.0-85048961138 OR 2-s2.0-84877691608 OR 2-s2.0-84974739765 OR 2-s2.0-33645960334 OR 2-s2.0-85097670709 OR 2-s2.0-33750951949 OR 2-s2.0-18944379387 OR 2-s2.0-85152211811 OR 2-s2.0-85149382143 OR 2-s2.0-84979917789 OR 2-s2.0-85123908483 OR 2-s2.0-85141892704 OR 2-s2.0-84962624055 OR 2-s2.0-84870893423 OR 2-s2.0-84999027826 OR 2-s2.0-85041407530 OR 2-s2.0-85078195276 OR 2-s2.0-85015811495 OR 2-s2.0-67649948710 OR 2-s2.0-34548856200 OR 2-s2.0-0035022536 OR 2-s2.0-0033736137 OR 2-s2.0-85062402027 OR 2-s2.0-0035002823 OR 2-s2.0-85131727015 OR 2-s2.0-80052003680 OR 2-s2.0-85095864874 OR 2-s2.0-85138152575 OR 2-s2.0-0035132807 OR 2-s2.0-85015359649 OR 2-s2.0-85138100456 OR 2-s2.0-85125234870 OR 2-s2.0-0034186101 OR 2-s2.0-79958289955 OR 2-s2.0-85087961945 OR 2-s2.0-85013720854 OR 2-s2.0-84886811106 OR 2-s2.0-80051981571 OR 2-s2.0-84874993259 OR 2-s2.0-84896310599 OR 2-s2.0-69649093196 OR 2-s2.0-85137719332 OR 2-s2.0-79952033330 OR 2-s2.0-34247183564 OR 2-s2.0-85063796395 OR 2-s2.0-84879191741 OR 2-s2.0-84857516171 OR 2-s2.0-77953731148 OR 2-s2.0-85119920301 OR 2-s2.0-85122473760 OR 2-s2.0-80052850827 OR 2-s2.0-85118325303 OR 2-s2.0-84867166849 OR 2-s2.0-85082504256 OR 2-s2.0-0034937048 OR 2-s2.0-84893283381 OR 2-s2.0-84866539356 OR 2-s2.0-85168003972 OR 2-s2.0-85026251268 OR 2-s2.0-85141843491 OR 2-s2.0-85063259484 OR 2-s2.0-85180104735 OR 2-s2.0-85107572409 OR 2-s2.0-85004011363 OR 2-s2.0-85012434763 OR 2-s2.0-85006164687 OR 2-s2.0-85106631393 OR 2-s2.0-85142226256 OR 2-s2.0-85071035980 OR 2-s2.0-17944386604 OR 2-s2.0-84883191694 OR 2-s2.0-85168140007 OR 2-s2.0-0028849296 OR 2-s2.0-85049775261 OR 2-s2.0-84919707604 OR 2-s2.0-84871482119 OR 2-s2.0-85126046322 OR 2-s2.0-84876289780 OR 2-s2.0-84952684290 OR 2-s2.0-85067984893 OR 2-s2.0-85160674840 OR 2-s2.0-84958971434 OR 2-s2.0-64249164660 OR 2-s2.0-84930982023 OR 2-s2.0-84861187410 OR 2-s2.0-85074923243 OR 2-s2.0-84922227587 OR 2-s2.0-0027947421 OR 2-s2.0-44649095716 OR 2-s2.0-85059374522 OR 2-s2.0-79955937971 OR 2-s2.0-85088776770 OR 2-s2.0-85030119749 OR 2-s2.0-85122457608 OR 2-s2.0-85159907765 OR 2-s2.0-84867120774 OR 2-s2.0-55949102994 OR 2-s2.0-80051795268 OR 2-s2.0-85160033148 OR 2-s2.0-84862616844 OR 2-s2.0-85138194107 OR 2-s2.0-84874328732 OR 2-s2.0-85125334062 OR 2-s2.0-84891681750 OR 2-s2.0-0028113928 OR 2-s2.0-85172279012 OR 2-s2.0-85013687613 OR 2-s2.0-85113782913 OR 2-s2.0-84856233436 OR 2-s2.0-84863395212 OR 2-s2.0-85085905565 OR 2-s2.0-85122655667 OR 2-s2.0-84907535343 OR 2-s2.0-85088940210 OR 2-s2.0-85020077381 OR 2-s2.0-85118229058 OR 2-s2.0-85178082804 OR 2-s2.0-84937419722 OR 2-s2.0-82455212983 OR 2-s2.0-85164592263 OR 2-s2.0-85080054743 OR 2-s2.0-84922216743 OR 2-s2.0-84888241918 OR 2-s2.0-0032801041 OR 2-s2.0-85091838918 OR 2-s2.0-27644480543 OR 2-s2.0-85140732450 OR 2-s2.0-85075647855 OR 2-s2.0-85013635475 OR 2-s2.0-84891722691 OR 2-s2.0-85160852495 OR 2-s2.0-84866179449 OR 2-s2.0-85176800660 OR 2-s2.0-57149096248 OR 2-s2.0-85178434193 OR 2-s2.0-0032932606 OR 2-s2.0-0029062333 OR 2-s2.0-85137931164 OR 2-s2.0-84897025367 OR 2-s2.0-84880923343 OR 2-s2.0-85002249030 OR 2-s2.0-84921885478 OR 2-s2.0-80051942883 OR 2-s2.0-85133741303 OR 2-s2.0-85156187935 ) AND ( LIMIT-TO ( SRCTYPE, “j” ) ) AND ( LIMIT-TO ( PUBSTAGE, “final” ) ) AND (LIMIT-TO ( DOCTYPE, “ar” ) ) AND ( EXCLUDE ( PUBYEAR, 2024 ) OR EXCLUDE ( PUBYEAR, 2025 ) )
5.	Sub	Voltage-Gated Potassium channel on therapeutic (3,628 records)	TITLE-ABS ( “ion* channel*” ) AND ( voltage AND gated AND potassium AND channel* ) AND ( “treatment*” OR “therapeutic*” ) AND NOT EID ( 2-s2.0-84927597176 OR 2-s2.0-84922010899 OR 2-s2.0-84900395214 OR 2-s2.0-85107710767 OR 2-s2.0-84962866792 OR 2-s2.0-33846673828 OR 2-s2.0-33947512419 OR 2-s2.0-85020991556 OR 2-s2.0-85013188047 OR 2-s2.0-84867125510 OR 2-s2.0-84891480845 OR 2-s2.0-84879845514 OR 2-s2.0-84942895036 OR 2-s2.0-84897059401 OR 2-s2.0-30844471383 OR 2-s2.0-85007352588 OR 2-s2.0-85039985438 OR 2-s2.0-0042536473 OR 2-s2.0-85021167561 OR 2-s2.0-79960698168 OR 2-s2.0-0037762690 OR 2-s2.0-85007368482 OR 2-s2.0-79955768829 OR 2-s2.0-84936928499 OR 2-s2.0-84951574682 OR 2-s2.0-70349335964 OR 2-s2.0-34447098802 OR 2-s2.0-85059541431 OR 2-s2.0-0030729279 OR 2-s2.0-77952309581 OR 2-s2.0-84883246797 OR 2-s2.0-85056496309 OR 2-s2.0-0025318574 OR 2-s2.0-85065493621 OR 2-s2.0-85060133773 OR 2-s2.0-0035313235 OR 2-s2.0-84962920731 OR 2-s2.0-84916207732 OR 2-s2.0-85046630803 OR 2-s2.0-84913549379 OR 2-s2.0-85007289400 OR 2-s2.0-36749015585 OR 2-s2.0-85066149701 OR 2-s2.0-79955558300 OR 2-s2.0-85073657423 OR 2-s2.0-85037690174 OR 2-s2.0-85074871955 OR 2-s2.0-84858161220 OR 2-s2.0-0023892933 OR 2-s2.0-79952262290 OR 2-s2.0-84981489482 OR 2-s2.0-84916201722 OR 2-s2.0-84890879754 OR 2-s2.0-84878393994 OR 2-s2.0-0037220090 OR 2-s2.0-84899860722 OR 2-s2.0-0036062999 OR 2-s2.0-84875794865 OR 2-s2.0-84958582085 OR 2-s2.0-84958242104 OR 2-s2.0-33750529349 OR 2-s2.0-85044159990 OR 2-s2.0-80054860325 OR 2-s2.0-84963751071 OR 2-s2.0-84893288265 OR 2-s2.0-85058928631 OR 2-s2.0-0031014473 OR 2-s2.0-84882705859 OR 2-s2.0-84880597393 OR 2-s2.0-84940413152 OR 2-s2.0-84877986414 OR 2-s2.0-0031030995 OR 2-s2.0-84883592437 OR 2-s2.0-33846264865 OR 2-s2.0-0030013925 OR 2-s2.0-84863777217 OR 2-s2.0-79959542489 OR 2-s2.0-58349114016 OR 2-s2.0-33745066809 OR 2-s2.0-84868126752 OR 2-s2.0-84866179386 OR 2-s2.0-84923819161 OR 2-s2.0-0032473367 OR 2-s2.0-70350379630 OR 2-s2.0-84925068443 OR 2-s2.0-33846188141 OR 2-s2.0-85078793098 OR 2-s2.0-84867899587 OR 2-s2.0-0036711450 OR 2-s2.0-84942886554 OR 2-s2.0-84921762521 OR 2-s2.0-84907515649 OR 2-s2.0-84864851318 OR 2-s2.0-0033066629 OR 2-s2.0-85082191409 OR 2-s2.0-84953297333 OR 2-s2.0-84945961579 OR 2-s2.0-84983788995 OR 2-s2.0-84871195861 OR 2-s2.0-85029213913 OR 2-s2.0-79955781475 OR 2-s2.0-84994959932 OR 2-s2.0-0033796095 OR 2-s2.0-84954285851 OR 2-s2.0-84896257270 OR 2-s2.0-85020011420 OR 2-s2.0-85058417690 OR 2-s2.0-85026610521 OR 2-s2.0-35648965716 OR 2-s2.0-84898772762 OR 2-s2.0-0034765967 OR 2-s2.0-85039868873 OR 2-s2.0-85083345183 OR 2-s2.0-84937808968 OR 2-s2.0-85071668624 OR 2-s2.0-85053042853 OR 2-s2.0-85007502838 OR 2-s2.0-85071777987 OR 2-s2.0-80052018704 OR 2-s2.0-84975781011 OR 2-s2.0-0027970905 OR 2-s2.0-85008474476 OR 2-s2.0-84994460502 OR 2-s2.0-84987851765 OR 2-s2.0-85070461451 OR 2-s2.0-79952082416 OR 2-s2.0-34547928925 OR 2-s2.0-85023744316 OR 2-s2.0-84889071254 OR 2-s2.0-77956830773 OR 2-s2.0-85112324505 OR 2-s2.0-84866179225 OR 2-s2.0-84922469457 OR 2-s2.0-0033016358 OR 2-s2.0-85118244016 OR 2-s2.0-85066492387 OR 2-s2.0-84869435941 OR 2-s2.0-85008702040 OR 2-s2.0-85041405270 OR 2-s2.0-85009442806 OR 2-s2.0-85065876846 OR 2-s2.0-84862023506 OR 2-s2.0-84930200978 OR 2-s2.0-84925340657 OR 2-s2.0-84895197157 OR 2-s2.0-84909583123 OR 2-s2.0-84931275456 OR 2-s2.0-85065229902 OR 2-s2.0-84911422091 OR 2-s2.0-79251630714 OR 2-s2.0-85097099229 OR 2-s2.0-84930644403 OR 2-s2.0-85131739568 OR 2-s2.0-84944047911 OR 2-s2.0-84952914192 OR 2-s2.0-84947802952 OR 2-s2.0-85111296029 OR 2-s2.0-84903791068 OR 2-s2.0-84942112073 OR 2-s2.0-0032886030 OR 2-s2.0-84930628571 OR 2-s2.0-84961684980 OR 2-s2.0-85043599838 OR 2-s2.0-84961218926 OR 2-s2.0-85130511821 OR 2-s2.0-0023296279 OR 2-s2.0-85093918416 OR 2-s2.0-50649102821 OR 2-s2.0-84867481677 OR 2-s2.0-84873371685 OR 2-s2.0-84866927563 OR 2-s2.0-34249790552 OR 2-s2.0-77953806557 OR 2-s2.0-84883057159 OR 2-s2.0-85018824585 OR 2-s2.0-85018255401 OR 2-s2.0-85048961138 OR 2-s2.0-84877691608 OR 2-s2.0-84974739765 OR 2-s2.0-33645960334 OR 2-s2.0-85097670709 OR 2-s2.0-33750951949 OR 2-s2.0-18944379387 OR 2-s2.0-85152211811 OR 2-s2.0-85149382143 OR 2-s2.0-84979917789 OR 2-s2.0-85123908483 OR 2-s2.0-85141892704 OR 2-s2.0-84962624055 OR 2-s2.0-84870893423 OR 2-s2.0-84999027826 OR 2-s2.0-85041407530 OR 2-s2.0-85078195276 OR 2-s2.0-85015811495 OR 2-s2.0-67649948710 OR 2-s2.0-34548856200 OR 2-s2.0-0035022536 OR 2-s2.0-0033736137 OR 2-s2.0-85062402027 OR 2-s2.0-0035002823 OR 2-s2.0-85131727015 OR 2-s2.0-80052003680 OR 2-s2.0-85095864874 OR 2-s2.0-85138152575 OR 2-s2.0-0035132807 OR 2-s2.0-85015359649 OR 2-s2.0-85138100456 OR 2-s2.0-85125234870 OR 2-s2.0-0034186101 OR 2-s2.0-79958289955 OR 2-s2.0-85087961945 OR 2-s2.0-85013720854 OR 2-s2.0-84886811106 OR 2-s2.0-80051981571 OR 2-s2.0-84874993259 OR 2-s2.0-84896310599 OR 2-s2.0-69649093196 OR 2-s2.0-85137719332 OR 2-s2.0-79952033330 OR 2-s2.0-34247183564 OR 2-s2.0-85063796395 OR 2-s2.0-84879191741 OR 2-s2.0-84857516171 OR 2-s2.0-77953731148 OR 2-s2.0-85119920301 OR 2-s2.0-85122473760 OR 2-s2.0-80052850827 OR 2-s2.0-85118325303 OR 2-s2.0-84867166849 OR 2-s2.0-85082504256 OR 2-s2.0-0034937048 OR 2-s2.0-84893283381 OR 2-s2.0-84866539356 OR 2-s2.0-85168003972 OR 2-s2.0-85026251268 OR 2-s2.0-85141843491 OR 2-s2.0-85063259484 OR 2-s2.0-85180104735 OR 2-s2.0-85107572409 OR 2-s2.0-85004011363 OR 2-s2.0-85012434763 OR 2-s2.0-85006164687 OR 2-s2.0-85106631393 OR 2-s2.0-85142226256 OR 2-s2.0-85071035980 OR 2-s2.0-17944386604 OR 2-s2.0-84883191694 OR 2-s2.0-85168140007 OR 2-s2.0-0028849296 OR 2-s2.0-85049775261 OR 2-s2.0-84919707604 OR 2-s2.0-84871482119 OR 2-s2.0-85126046322 OR 2-s2.0-84876289780 OR 2-s2.0-84952684290 OR 2-s2.0-85067984893 OR 2-s2.0-85160674840 OR 2-s2.0-84958971434 OR 2-s2.0-64249164660 OR 2-s2.0-84930982023 OR 2-s2.0-84861187410 OR 2-s2.0-85074923243 OR 2-s2.0-84922227587 OR 2-s2.0-0027947421 OR 2-s2.0-44649095716 OR 2-s2.0-85059374522 OR 2-s2.0-79955937971 OR 2-s2.0-85088776770 OR 2-s2.0-85030119749 OR 2-s2.0-85122457608 OR 2-s2.0-85159907765 OR 2-s2.0-84867120774 OR 2-s2.0-55949102994 OR 2-s2.0-80051795268 OR 2-s2.0-85160033148 OR 2-s2.0-84862616844 OR 2-s2.0-85138194107 OR 2-s2.0-84874328732 OR 2-s2.0-85125334062 OR 2-s2.0-84891681750 OR 2-s2.0-0028113928 OR 2-s2.0-85172279012 OR 2-s2.0-85013687613 OR 2-s2.0-85113782913 OR 2-s2.0-84856233436 OR 2-s2.0-84863395212 OR 2-s2.0-85085905565 OR 2-s2.0-85122655667 OR 2-s2.0-84907535343 OR 2-s2.0-85088940210 OR 2-s2.0-85020077381 OR 2-s2.0-85118229058 OR 2-s2.0-85178082804 OR 2-s2.0-84937419722 OR 2-s2.0-82455212983 OR 2-s2.0-85164592263 OR 2-s2.0-85080054743 OR 2-s2.0-84922216743 OR 2-s2.0-84888241918 OR 2-s2.0-0032801041 OR 2-s2.0-85091838918 OR 2-s2.0-27644480543 OR 2-s2.0-85140732450 OR 2-s2.0-85075647855 OR 2-s2.0-85013635475 OR 2-s2.0-84891722691 OR 2-s2.0-85160852495 OR 2-s2.0-84866179449 OR 2-s2.0-85176800660 OR 2-s2.0-57149096248 OR 2-s2.0-85178434193 OR 2-s2.0-0032932606 OR 2-s2.0-0029062333 OR 2-s2.0-85137931164 OR 2-s2.0-84897025367 OR 2-s2.0-84880923343 OR 2-s2.0-85002249030 OR 2-s2.0-84921885478 OR 2-s2.0-80051942883 OR 2-s2.0-85133741303 OR 2-s2.0-85156187935 ) AND ( LIMIT-TO ( SRCTYPE, “j” ) ) AND ( LIMIT-TO ( PUBSTAGE, “final” ) ) AND ( LIMIT-TO ( DOCTYPE, “ar” ) ) AND ( EXCLUDE ( PUBYEAR, 2024 ) OR EXCLUDE ( PUBYEAR, 2025 ) )
6.	Sub	Voltage-Gated Chloride channel on therapeutic (1,140 records)	TITLE-ABS ( “ion* channel*” ) AND ( voltage AND gated AND chloride AND channel* ) AND ( “treatment*” OR “therapeutic*” ) AND NOT EID ( 2-s2.0-84927597176 OR 2-s2.0-84922010899 OR 2-s2.0-84900395214 OR 2-s2.0-85107710767 OR 2-s2.0-84962866792 OR 2-s2.0-33846673828 OR 2-s2.0-33947512419 OR 2-s2.0-85020991556 OR 2-s2.0-85013188047 OR 2-s2.0-84867125510 OR 2-s2.0-84891480845 OR 2-s2.0-84879845514 OR 2-s2.0-84942895036 OR 2-s2.0-84897059401 OR 2-s2.0-30844471383 OR 2-s2.0-85007352588 OR 2-s2.0-85039985438 OR 2-s2.0-0042536473 OR 2-s2.0-85021167561 OR 2-s2.0-79960698168 OR 2-s2.0-0037762690 OR 2-s2.0-85007368482 OR 2-s2.0-79955768829 OR 2-s2.0-84936928499 OR 2-s2.0-84951574682 OR 2-s2.0-70349335964 OR 2-s2.0-34447098802 OR 2-s2.0-85059541431 OR 2-s2.0-0030729279 OR 2-s2.0-77952309581 OR 2-s2.0-84883246797 OR 2-s2.0-85056496309 OR 2-s2.0-0025318574 OR 2-s2.0-85065493621 OR 2-s2.0-85060133773 OR 2-s2.0-0035313235 OR 2-s2.0-84962920731 OR 2-s2.0-84916207732 OR 2-s2.0-85046630803 OR 2-s2.0-84913549379 OR 2-s2.0-85007289400 OR 2-s2.0-36749015585 OR 2-s2.0-85066149701 OR 2-s2.0-79955558300 OR 2-s2.0-85073657423 OR 2-s2.0-85037690174 OR 2-s2.0-85074871955 OR 2-s2.0-84858161220 OR 2-s2.0-0023892933 OR 2-s2.0-79952262290 OR 2-s2.0-84981489482 OR 2-s2.0-84916201722 OR 2-s2.0-84890879754 OR 2-s2.0-84878393994 OR 2-s2.0-0037220090 OR 2-s2.0-84899860722 OR 2-s2.0-0036062999 OR 2-s2.0-84875794865 OR 2-s2.0-84958582085 OR 2-s2.0-84958242104 OR 2-s2.0-33750529349 OR 2-s2.0-85044159990 OR 2-s2.0-80054860325 OR 2-s2.0-84963751071 OR 2-s2.0-84893288265 OR 2-s2.0-85058928631 OR 2-s2.0-0031014473 OR 2-s2.0-84882705859 OR 2-s2.0-84880597393 OR 2-s2.0-84940413152 OR 2-s2.0-84877986414 OR 2-s2.0-0031030995 OR 2-s2.0-84883592437 OR 2-s2.0-33846264865 OR 2-s2.0-0030013925 OR 2-s2.0-84863777217 OR 2-s2.0-79959542489 OR 2-s2.0-58349114016 OR 2-s2.0-33745066809 OR 2-s2.0-84868126752 OR 2-s2.0-84866179386 OR 2-s2.0-84923819161 OR 2-s2.0-0032473367 OR 2-s2.0-70350379630 OR 2-s2.0-84925068443 OR 2-s2.0-33846188141 OR 2-s2.0-85078793098 OR 2-s2.0-84867899587 OR 2-s2.0-0036711450 OR 2-s2.0-84942886554 OR 2-s2.0-84921762521 OR 2-s2.0-84907515649 OR 2-s2.0-84864851318 OR 2-s2.0-0033066629 OR 2-s2.0-85082191409 OR 2-s2.0-84953297333 OR 2-s2.0-84945961579 OR 2-s2.0-84983788995 OR 2-s2.0-84871195861 OR 2-s2.0-85029213913 OR 2-s2.0-79955781475 OR 2-s2.0-84994959932 OR 2-s2.0-0033796095 OR 2-s2.0-84954285851 OR 2-s2.0-84896257270 OR 2-s2.0-85020011420 OR 2-s2.0-85058417690 OR 2-s2.0-85026610521 OR 2-s2.0-35648965716 OR 2-s2.0-84898772762 OR 2-s2.0-0034765967 OR 2-s2.0-85039868873 OR 2-s2.0-85083345183 OR 2-s2.0-84937808968 OR 2-s2.0-85071668624 OR 2-s2.0-85053042853 OR 2-s2.0-85007502838 OR 2-s2.0-85071777987 OR 2-s2.0-80052018704 OR 2-s2.0-84975781011 OR 2-s2.0-0027970905 OR 2-s2.0-85008474476 OR 2-s2.0-84994460502 OR 2-s2.0-84987851765 OR 2-s2.0-85070461451 OR 2-s2.0-79952082416 OR 2-s2.0-34547928925 OR 2-s2.0-85023744316 OR 2-s2.0-84889071254 OR 2-s2.0-77956830773 OR 2-s2.0-85112324505 OR 2-s2.0-84866179225 OR 2-s2.0-84922469457 OR 2-s2.0-0033016358 OR 2-s2.0-85118244016 OR 2-s2.0-85066492387 OR 2-s2.0-84869435941 OR 2-s2.0-85008702040 OR 2-s2.0-85041405270 OR 2-s2.0-85009442806 OR 2-s2.0-85065876846 OR 2-s2.0-84862023506 OR 2-s2.0-84930200978 OR 2-s2.0-84925340657 OR 2-s2.0-84895197157 OR 2-s2.0-84909583123 OR 2-s2.0-84931275456 OR 2-s2.0-85065229902 OR 2-s2.0-84911422091 OR 2-s2.0-79251630714 OR 2-s2.0-85097099229 OR 2-s2.0-84930644403 OR 2-s2.0-85131739568 OR 2-s2.0-84944047911 OR 2-s2.0-84952914192 OR 2-s2.0-84947802952 OR 2-s2.0-85111296029 OR 2-s2.0-84903791068 OR 2-s2.0-84942112073 OR 2-s2.0-0032886030 OR 2-s2.0-84930628571 OR 2-s2.0-84961684980 OR 2-s2.0-85043599838 OR 2-s2.0-84961218926 OR 2-s2.0-85130511821 OR 2-s2.0-0023296279 OR 2-s2.0-85093918416 OR 2-s2.0-50649102821 OR 2-s2.0-84867481677 OR 2-s2.0-84873371685 OR 2-s2.0-84866927563 OR 2-s2.0-34249790552 OR 2-s2.0-77953806557 OR 2-s2.0-84883057159 OR 2-s2.0-85018824585 OR 2-s2.0-85018255401 OR 2-s2.0-85048961138 OR 2-s2.0-84877691608 OR 2-s2.0-84974739765 OR 2-s2.0-33645960334 OR 2-s2.0-85097670709 OR 2-s2.0-33750951949 OR 2-s2.0-18944379387 OR 2-s2.0-85152211811 OR 2-s2.0-85149382143 OR 2-s2.0-84979917789 OR 2-s2.0-85123908483 OR 2-s2.0-85141892704 OR 2-s2.0-84962624055 OR 2-s2.0-84870893423 OR 2-s2.0-84999027826 OR 2-s2.0-85041407530 OR 2-s2.0-85078195276 OR 2-s2.0-85015811495 OR 2-s2.0-67649948710 OR 2-s2.0-34548856200 OR 2-s2.0-0035022536 OR 2-s2.0-0033736137 OR 2-s2.0-85062402027 OR 2-s2.0-0035002823 OR 2-s2.0-85131727015 OR 2-s2.0-80052003680 OR 2-s2.0-85095864874 OR 2-s2.0-85138152575 OR 2-s2.0-0035132807 OR 2-s2.0-85015359649 OR 2-s2.0-85138100456 OR 2-s2.0-85125234870 OR 2-s2.0-0034186101 OR 2-s2.0-79958289955 OR 2-s2.0-85087961945 OR 2-s2.0-85013720854 OR 2-s2.0-84886811106 OR 2-s2.0-80051981571 OR 2-s2.0-84874993259 OR 2-s2.0-84896310599 OR 2-s2.0-69649093196 OR 2-s2.0-85137719332 OR 2-s2.0-79952033330 OR 2-s2.0-34247183564 OR 2-s2.0-85063796395 OR 2-s2.0-84879191741 OR 2-s2.0-84857516171 OR 2-s2.0-77953731148 OR 2-s2.0-85119920301 OR 2-s2.0-85122473760 OR 2-s2.0-80052850827 OR 2-s2.0-85118325303 OR 2-s2.0-84867166849 OR 2-s2.0-85082504256 OR 2-s2.0-0034937048 OR 2-s2.0-84893283381 OR 2-s2.0-84866539356 OR 2-s2.0-85168003972 OR 2-s2.0-85026251268 OR 2-s2.0-85141843491 OR 2-s2.0-85063259484 OR 2-s2.0-85180104735 OR 2-s2.0-85107572409 OR 2-s2.0-85004011363 OR 2-s2.0-85012434763 OR 2-s2.0-85006164687 OR 2-s2.0-85106631393 OR 2-s2.0-85142226256 OR 2-s2.0-85071035980 OR 2-s2.0-17944386604 OR 2-s2.0-84883191694 OR 2-s2.0-85168140007 OR 2-s2.0-0028849296 OR 2-s2.0-85049775261 OR 2-s2.0-84919707604 OR 2-s2.0-84871482119 OR 2-s2.0-85126046322 OR 2-s2.0-84876289780 OR 2-s2.0-84952684290 OR 2-s2.0-85067984893 OR 2-s2.0-85160674840 OR 2-s2.0-84958971434 OR 2-s2.0-64249164660 OR 2-s2.0-84930982023 OR 2-s2.0-84861187410 OR 2-s2.0-85074923243 OR 2-s2.0-84922227587 OR 2-s2.0-0027947421 OR 2-s2.0-44649095716 OR 2-s2.0-85059374522 OR 2-s2.0-79955937971 OR 2-s2.0-85088776770 OR 2-s2.0-85030119749 OR 2-s2.0-85122457608 OR 2-s2.0-85159907765 OR 2-s2.0-84867120774 OR 2-s2.0-55949102994 OR 2-s2.0-80051795268 OR 2-s2.0-85160033148 OR 2-s2.0-84862616844 OR 2-s2.0-85138194107 OR 2-s2.0-84874328732 OR 2-s2.0-85125334062 OR 2-s2.0-84891681750 OR 2-s2.0-0028113928 OR 2-s2.0-85172279012 OR 2-s2.0-85013687613 OR 2-s2.0-85113782913 OR 2-s2.0-84856233436 OR 2-s2.0-84863395212 OR 2-s2.0-85085905565 OR 2-s2.0-85122655667 OR 2-s2.0-84907535343 OR 2-s2.0-85088940210 OR 2-s2.0-85020077381 OR 2-s2.0-85118229058 OR 2-s2.0-85178082804 OR 2-s2.0-84937419722 OR 2-s2.0-82455212983 OR 2-s2.0-85164592263 OR 2-s2.0-85080054743 OR 2-s2.0-84922216743 OR 2-s2.0-84888241918 OR 2-s2.0-0032801041 OR 2-s2.0-85091838918 OR 2-s2.0-27644480543 OR 2-s2.0-85140732450 OR 2-s2.0-85075647855 OR 2-s2.0-85013635475 OR 2-s2.0-84891722691 OR 2-s2.0-85160852495 OR 2-s2.0-84866179449 OR 2-s2.0-85176800660 OR 2-s2.0-57149096248 OR 2-s2.0-85178434193 OR 2-s2.0-0032932606 OR 2-s2.0-0029062333 OR 2-s2.0-85137931164 OR 2-s2.0-84897025367 OR 2-s2.0-84880923343 OR 2-s2.0-85002249030 OR 2-s2.0-84921885478 OR 2-s2.0-80051942883 OR 2-s2.0-85133741303 OR 2-s2.0-85156187935 ) AND ( LIMIT-TO ( SRCTYPE,”j” ) ) AND ( LIMIT-TO ( PUBSTAGE,”final” ) ) AND ( LIMIT-TO ( DOCTYPE,”ar” ) ) AND ( EXCLUDE ( PUBYEAR,2024) OR EXCLUDE ( PUBYEAR,2025) )
7.	Sub	Voltage-Gated Calcium channel on therapeutic (3,917 records)	TITLE-ABS ( “ion* channel*” ) AND ( voltage AND gated AND calcium AND channel* ) AND ( “treatment*” OR “therapeutic*” ) AND NOT EID ( 2-s2.0-84927597176 OR 2-s2.0-84922010899 OR 2-s2.0-84900395214 OR 2-s2.0-85107710767 OR 2-s2.0-84962866792 OR 2-s2.0-33846673828 OR 2-s2.0-33947512419 OR 2-s2.0-85020991556 OR 2-s2.0-85013188047 OR 2-s2.0-84867125510 OR 2-s2.0-84891480845 OR 2-s2.0-84879845514 OR 2-s2.0-84942895036 OR 2-s2.0-84897059401 OR 2-s2.0-30844471383 OR 2-s2.0-85007352588 OR 2-s2.0-85039985438 OR 2-s2.0-0042536473 OR 2-s2.0-85021167561 OR 2-s2.0-79960698168 OR 2-s2.0-0037762690 OR 2-s2.0-85007368482 OR 2-s2.0-79955768829 OR 2-s2.0-84936928499 OR 2-s2.0-84951574682 OR 2-s2.0-70349335964 OR 2-s2.0-34447098802 OR 2-s2.0-85059541431 OR 2-s2.0-0030729279 OR 2-s2.0-77952309581 OR 2-s2.0-84883246797 OR 2-s2.0-85056496309 OR 2-s2.0-0025318574 OR 2-s2.0-85065493621 OR 2-s2.0-85060133773 OR 2-s2.0-0035313235 OR 2-s2.0-84962920731 OR 2-s2.0-84916207732 OR 2-s2.0-85046630803 OR 2-s2.0-84913549379 OR 2-s2.0-85007289400 OR 2-s2.0-36749015585 OR 2-s2.0-85066149701 OR 2-s2.0-79955558300 OR 2-s2.0-85073657423 OR 2-s2.0-85037690174 OR 2-s2.0-85074871955 OR 2-s2.0-84858161220 OR 2-s2.0-0023892933 OR 2-s2.0-79952262290 OR 2-s2.0-84981489482 OR 2-s2.0-84916201722 OR 2-s2.0-84890879754 OR 2-s2.0-84878393994 OR 2-s2.0-0037220090 OR 2-s2.0-84899860722 OR 2-s2.0-0036062999 OR 2-s2.0-84875794865 OR 2-s2.0-84958582085 OR 2-s2.0-84958242104 OR 2-s2.0-33750529349 OR 2-s2.0-85044159990 OR 2-s2.0-80054860325 OR 2-s2.0-84963751071 OR 2-s2.0-84893288265 OR 2-s2.0-85058928631 OR 2-s2.0-0031014473 OR 2-s2.0-84882705859 OR 2-s2.0-84880597393 OR 2-s2.0-84940413152 OR 2-s2.0-84877986414 OR 2-s2.0-0031030995 OR 2-s2.0-84883592437 OR 2-s2.0-33846264865 OR 2-s2.0-0030013925 OR 2-s2.0-84863777217 OR 2-s2.0-79959542489 OR 2-s2.0-58349114016 OR 2-s2.0-33745066809 OR 2-s2.0-84868126752 OR 2-s2.0-84866179386 OR 2-s2.0-84923819161 OR 2-s2.0-0032473367 OR 2-s2.0-70350379630 OR 2-s2.0-84925068443 OR 2-s2.0-33846188141 OR 2-s2.0-85078793098 OR 2-s2.0-84867899587 OR 2-s2.0-0036711450 OR 2-s2.0-84942886554 OR 2-s2.0-84921762521 OR 2-s2.0-84907515649 OR 2-s2.0-84864851318 OR 2-s2.0-0033066629 OR 2-s2.0-85082191409 OR 2-s2.0-84953297333 OR 2-s2.0-84945961579 OR 2-s2.0-84983788995 OR 2-s2.0-84871195861 OR 2-s2.0-85029213913 OR 2-s2.0-79955781475 OR 2-s2.0-84994959932 OR 2-s2.0-0033796095 OR 2-s2.0-84954285851 OR 2-s2.0-84896257270 OR 2-s2.0-85020011420 OR 2-s2.0-85058417690 OR 2-s2.0-85026610521 OR 2-s2.0-35648965716 OR 2-s2.0-84898772762 OR 2-s2.0-0034765967 OR 2-s2.0-85039868873 OR 2-s2.0-85083345183 OR 2-s2.0-84937808968 OR 2-s2.0-85071668624 OR 2-s2.0-85053042853 OR 2-s2.0-85007502838 OR 2-s2.0-85071777987 OR 2-s2.0-80052018704 OR 2-s2.0-84975781011 OR 2-s2.0-0027970905 OR 2-s2.0-85008474476 OR 2-s2.0-84994460502 OR 2-s2.0-84987851765 OR 2-s2.0-85070461451 OR 2-s2.0-79952082416 OR 2-s2.0-34547928925 OR 2-s2.0-85023744316 OR 2-s2.0-84889071254 OR 2-s2.0-77956830773 OR 2-s2.0-85112324505 OR 2-s2.0-84866179225 OR 2-s2.0-84922469457 OR 2-s2.0-0033016358 OR 2-s2.0-85118244016 OR 2-s2.0-85066492387 OR 2-s2.0-84869435941 OR 2-s2.0-85008702040 OR 2-s2.0-85041405270 OR 2-s2.0-85009442806 OR 2-s2.0-85065876846 OR 2-s2.0-84862023506 OR 2-s2.0-84930200978 OR 2-s2.0-84925340657 OR 2-s2.0-84895197157 OR 2-s2.0-84909583123 OR 2-s2.0-84931275456 OR 2-s2.0-85065229902 OR 2-s2.0-84911422091 OR 2-s2.0-79251630714 OR 2-s2.0-85097099229 OR 2-s2.0-84930644403 OR 2-s2.0-85131739568 OR 2-s2.0-84944047911 OR 2-s2.0-84952914192 OR 2-s2.0-84947802952 OR 2-s2.0-85111296029 OR 2-s2.0-84903791068 OR 2-s2.0-84942112073 OR 2-s2.0-0032886030 OR 2-s2.0-84930628571 OR 2-s2.0-84961684980 OR 2-s2.0-85043599838 OR 2-s2.0-84961218926 OR 2-s2.0-85130511821 OR 2-s2.0-0023296279 OR 2-s2.0-85093918416 OR 2-s2.0-50649102821 OR 2-s2.0-84867481677 OR 2-s2.0-84873371685 OR 2-s2.0-84866927563 OR 2-s2.0-34249790552 OR 2-s2.0-77953806557 OR 2-s2.0-84883057159 OR 2-s2.0-85018824585 OR 2-s2.0-85018255401 OR 2-s2.0-85048961138 OR 2-s2.0-84877691608 OR 2-s2.0-84974739765 OR 2-s2.0-33645960334 OR 2-s2.0-85097670709 OR 2-s2.0-33750951949 OR 2-s2.0-18944379387 OR 2-s2.0-85152211811 OR 2-s2.0-85149382143 OR 2-s2.0-84979917789 OR 2-s2.0-85123908483 OR 2-s2.0-85141892704 OR 2-s2.0-84962624055 OR 2-s2.0-84870893423 OR 2-s2.0-84999027826 OR 2-s2.0-85041407530 OR 2-s2.0-85078195276 OR 2-s2.0-85015811495 OR 2-s2.0-67649948710 OR 2-s2.0-34548856200 OR 2-s2.0-0035022536 OR 2-s2.0-0033736137 OR 2-s2.0-85062402027 OR 2-s2.0-0035002823 OR 2-s2.0-85131727015 OR 2-s2.0-80052003680 OR 2-s2.0-85095864874 OR 2-s2.0-85138152575 OR 2-s2.0-0035132807 OR 2-s2.0-85015359649 OR 2-s2.0-85138100456 OR 2-s2.0-85125234870 OR 2-s2.0-0034186101 OR 2-s2.0-79958289955 OR 2-s2.0-85087961945 OR 2-s2.0-85013720854 OR 2-s2.0-84886811106 OR 2-s2.0-80051981571 OR 2-s2.0-84874993259 OR 2-s2.0-84896310599 OR 2-s2.0-69649093196 OR 2-s2.0-85137719332 OR 2-s2.0-79952033330 OR 2-s2.0-34247183564 OR 2-s2.0-85063796395 OR 2-s2.0-84879191741 OR 2-s2.0-84857516171 OR 2-s2.0-77953731148 OR 2-s2.0-85119920301 OR 2-s2.0-85122473760 OR 2-s2.0-80052850827 OR 2-s2.0-85118325303 OR 2-s2.0-84867166849 OR 2-s2.0-85082504256 OR 2-s2.0-0034937048 OR 2-s2.0-84893283381 OR 2-s2.0-84866539356 OR 2-s2.0-85168003972 OR 2-s2.0-85026251268 OR 2-s2.0-85141843491 OR 2-s2.0-85063259484 OR 2-s2.0-85180104735 OR 2-s2.0-85107572409 OR 2-s2.0-85004011363 OR 2-s2.0-85012434763 OR 2-s2.0-85006164687 OR 2-s2.0-85106631393 OR 2-s2.0-85142226256 OR 2-s2.0-85071035980 OR 2-s2.0-17944386604 OR 2-s2.0-84883191694 OR 2-s2.0-85168140007 OR 2-s2.0-0028849296 OR 2-s2.0-85049775261 OR 2-s2.0-84919707604 OR 2-s2.0-84871482119 OR 2-s2.0-85126046322 OR 2-s2.0-84876289780 OR 2-s2.0-84952684290 OR 2-s2.0-85067984893 OR 2-s2.0-85160674840 OR 2-s2.0-84958971434 OR 2-s2.0-64249164660 OR 2-s2.0-84930982023 OR 2-s2.0-84861187410 OR 2-s2.0-85074923243 OR 2-s2.0-84922227587 OR 2-s2.0-0027947421 OR 2-s2.0-44649095716 OR 2-s2.0-85059374522 OR 2-s2.0-79955937971 OR 2-s2.0-85088776770 OR 2-s2.0-85030119749 OR 2-s2.0-85122457608 OR 2-s2.0-85159907765 OR 2-s2.0-84867120774 OR 2-s2.0-55949102994 OR 2-s2.0-80051795268 OR 2-s2.0-85160033148 OR 2-s2.0-84862616844 OR 2-s2.0-85138194107 OR 2-s2.0-84874328732 OR 2-s2.0-85125334062 OR 2-s2.0-84891681750 OR 2-s2.0-0028113928 OR 2-s2.0-85172279012 OR 2-s2.0-85013687613 OR 2-s2.0-85113782913 OR 2-s2.0-84856233436 OR 2-s2.0-84863395212 OR 2-s2.0-85085905565 OR 2-s2.0-85122655667 OR 2-s2.0-84907535343 OR 2-s2.0-85088940210 OR 2-s2.0-85020077381 OR 2-s2.0-85118229058 OR 2-s2.0-85178082804 OR 2-s2.0-84937419722 OR 2-s2.0-82455212983 OR 2-s2.0-85164592263 OR 2-s2.0-85080054743 OR 2-s2.0-84922216743 OR 2-s2.0-84888241918 OR 2-s2.0-0032801041 OR 2-s2.0-85091838918 OR 2-s2.0-27644480543 OR 2-s2.0-85140732450 OR 2-s2.0-85075647855 OR 2-s2.0-85013635475 OR 2-s2.0-84891722691 OR 2-s2.0-85160852495 OR 2-s2.0-84866179449 OR 2-s2.0-85176800660 OR 2-s2.0-57149096248 OR 2-s2.0-85178434193 OR 2-s2.0-0032932606 OR 2-s2.0-0029062333 OR 2-s2.0-85137931164 OR 2-s2.0-84897025367 OR 2-s2.0-84880923343 OR 2-s2.0-85002249030 OR 2-s2.0-84921885478 OR 2-s2.0-80051942883 OR 2-s2.0-85133741303 OR 2-s2.0-85156187935 ) AND ( LIMIT-TO ( SRCTYPE, “j” ) ) AND ( LIMIT-TO ( PUBSTAGE, “final” ) ) AND ( LIMIT-TO ( DOCTYPE, “ar” ) ) AND ( EXCLUDE ( PUBYEAR, 2024 ) OR EXCLUDE ( PUBYEAR, 2025 ) )
8.	Sub	VGSC as Treatment in genetic disease (1,168 records)	TITLE-ABS ( “ion* channel*” ) AND ( voltage AND gated AND sodium AND channel* ) AND ( “treatment*” OR “therapeutic*” ) AND ( “genetic*” AND “disease*” ) AND NOT EID ( 2-s2.0-84927597176 OR 2-s2.0-84922010899 OR 2-s2.0-84900395214 OR 2-s2.0-85107710767 OR 2-s2.0-84962866792 OR 2-s2.0-33846673828 OR 2-s2.0-33947512419 OR 2-s2.0-85020991556 OR 2-s2.0-85013188047 OR 2-s2.0-84867125510 OR 2-s2.0-84891480845 OR 2-s2.0-84879845514 OR 2-s2.0-84942895036 OR 2-s2.0-84897059401 OR 2-s2.0-30844471383 OR 2-s2.0-85007352588 OR 2-s2.0-85039985438 OR 2-s2.0-0042536473 OR 2-s2.0-85021167561 OR 2-s2.0-79960698168 OR 2-s2.0-0037762690 OR 2-s2.0-85007368482 OR 2-s2.0-79955768829 OR 2-s2.0-84936928499 OR 2-s2.0-84951574682 OR 2-s2.0-70349335964 OR 2-s2.0-34447098802 OR 2-s2.0-85059541431 OR 2-s2.0-0030729279 OR 2-s2.0-77952309581 OR 2-s2.0-84883246797 OR 2-s2.0-85056496309 OR 2-s2.0-0025318574 OR 2-s2.0-85065493621 OR 2-s2.0-85060133773 OR 2-s2.0-0035313235 OR 2-s2.0-84962920731 OR 2-s2.0-84916207732 OR 2-s2.0-85046630803 OR 2-s2.0-84913549379 OR 2-s2.0-85007289400 OR 2-s2.0-36749015585 OR 2-s2.0-85066149701 OR 2-s2.0-79955558300 OR 2-s2.0-85073657423 OR 2-s2.0-85037690174 OR 2-s2.0-85074871955 OR 2-s2.0-84858161220 OR 2-s2.0-0023892933 OR 2-s2.0-79952262290 OR 2-s2.0-84981489482 OR 2-s2.0-84916201722 OR 2-s2.0-84890879754 OR 2-s2.0-84878393994 OR 2-s2.0-0037220090 OR 2-s2.0-84899860722 OR 2-s2.0-0036062999 OR 2-s2.0-84875794865 OR 2-s2.0-84958582085 OR 2-s2.0-84958242104 OR 2-s2.0-33750529349 OR 2-s2.0-85044159990 OR 2-s2.0-80054860325 OR 2-s2.0-84963751071 OR 2-s2.0-84893288265 OR 2-s2.0-85058928631 OR 2-s2.0-0031014473 OR 2-s2.0-84882705859 OR 2-s2.0-84880597393 OR 2-s2.0-84940413152 OR 2-s2.0-84877986414 OR 2-s2.0-0031030995 OR 2-s2.0-84883592437 OR 2-s2.0-33846264865 OR 2-s2.0-0030013925 OR 2-s2.0-84863777217 OR 2-s2.0-79959542489 OR 2-s2.0-58349114016 OR 2-s2.0-33745066809 OR 2-s2.0-84868126752 OR 2-s2.0-84866179386 OR 2-s2.0-84923819161 OR 2-s2.0-0032473367 OR 2-s2.0-70350379630 OR 2-s2.0-84925068443 OR 2-s2.0-33846188141 OR 2-s2.0-85078793098 OR 2-s2.0-84867899587 OR 2-s2.0-0036711450 OR 2-s2.0-84942886554 OR 2-s2.0-84921762521 OR 2-s2.0-84907515649 OR 2-s2.0-84864851318 OR 2-s2.0-0033066629 OR 2-s2.0-85082191409 OR 2-s2.0-84953297333 OR 2-s2.0-84945961579 OR 2-s2.0-84983788995 OR 2-s2.0-84871195861 OR 2-s2.0-85029213913 OR 2-s2.0-79955781475 OR 2-s2.0-84994959932 OR 2-s2.0-0033796095 OR 2-s2.0-84954285851 OR 2-s2.0-84896257270 OR 2-s2.0-85020011420 OR 2-s2.0-85058417690 OR 2-s2.0-85026610521 OR 2-s2.0-35648965716 OR 2-s2.0-84898772762 OR 2-s2.0-0034765967 OR 2-s2.0-85039868873 OR 2-s2.0-85083345183 OR 2-s2.0-84937808968 OR 2-s2.0-85071668624 OR 2-s2.0-85053042853 OR 2-s2.0-85007502838 OR 2-s2.0-85071777987 OR 2-s2.0-80052018704 OR 2-s2.0-84975781011 OR 2-s2.0-0027970905 OR 2-s2.0-85008474476 OR 2-s2.0-84994460502 OR 2-s2.0-84987851765 OR 2-s2.0-85070461451 OR 2-s2.0-79952082416 OR 2-s2.0-34547928925 OR 2-s2.0-85023744316 OR 2-s2.0-84889071254 OR 2-s2.0-77956830773 OR 2-s2.0-85112324505 OR 2-s2.0-84866179225 OR 2-s2.0-84922469457 OR 2-s2.0-0033016358 OR 2-s2.0-85118244016 OR 2-s2.0-85066492387 OR 2-s2.0-84869435941 OR 2-s2.0-85008702040 OR 2-s2.0-85041405270 OR 2-s2.0-85009442806 OR 2-s2.0-85065876846 OR 2-s2.0-84862023506 OR 2-s2.0-84930200978 OR 2-s2.0-84925340657 OR 2-s2.0-84895197157 OR 2-s2.0-84909583123 OR 2-s2.0-84931275456 OR 2-s2.0-85065229902 OR 2-s2.0-84911422091 OR 2-s2.0-79251630714 OR 2-s2.0-85097099229 OR 2-s2.0-84930644403 OR 2-s2.0-85131739568 OR 2-s2.0-84944047911 OR 2-s2.0-84952914192 OR 2-s2.0-84947802952 OR 2-s2.0-85111296029 OR 2-s2.0-84903791068 OR 2-s2.0-84942112073 OR 2-s2.0-0032886030 OR 2-s2.0-84930628571 OR 2-s2.0-84961684980 OR 2-s2.0-85043599838 OR 2-s2.0-84961218926 OR 2-s2.0-85130511821 OR 2-s2.0-0023296279 OR 2-s2.0-85093918416 OR 2-s2.0-50649102821 OR 2-s2.0-84867481677 OR 2-s2.0-84873371685 OR 2-s2.0-84866927563 OR 2-s2.0-34249790552 OR 2-s2.0-77953806557 OR 2-s2.0-84883057159 OR 2-s2.0-85018824585 OR 2-s2.0-85018255401 OR 2-s2.0-85048961138 OR 2-s2.0-84877691608 OR 2-s2.0-84974739765 OR 2-s2.0-33645960334 OR 2-s2.0-85097670709 OR 2-s2.0-33750951949 OR 2-s2.0-18944379387 OR 2-s2.0-85152211811 OR 2-s2.0-85149382143 OR 2-s2.0-84979917789 OR 2-s2.0-85123908483 OR 2-s2.0-85141892704 OR 2-s2.0-84962624055 OR 2-s2.0-84870893423 OR 2-s2.0-84999027826 OR 2-s2.0-85041407530 OR 2-s2.0-85078195276 OR 2-s2.0-85015811495 OR 2-s2.0-67649948710 OR 2-s2.0-34548856200 OR 2-s2.0-0035022536 OR 2-s2.0-0033736137 OR 2-s2.0-85062402027 OR 2-s2.0-0035002823 OR 2-s2.0-85131727015 OR 2-s2.0-80052003680 OR 2-s2.0-85095864874 OR 2-s2.0-85138152575 OR 2-s2.0-0035132807 OR 2-s2.0-85015359649 OR 2-s2.0-85138100456 OR 2-s2.0-85125234870 OR 2-s2.0-0034186101 OR 2-s2.0-79958289955 OR 2-s2.0-85087961945 OR 2-s2.0-85013720854 OR 2-s2.0-84886811106 OR 2-s2.0-80051981571 OR 2-s2.0-84874993259 OR 2-s2.0-84896310599 OR 2-s2.0-69649093196 OR 2-s2.0-85137719332 OR 2-s2.0-79952033330 OR 2-s2.0-34247183564 OR 2-s2.0-85063796395 OR 2-s2.0-84879191741 OR 2-s2.0-84857516171 OR 2-s2.0-77953731148 OR 2-s2.0-85119920301 OR 2-s2.0-85122473760 OR 2-s2.0-80052850827 OR 2-s2.0-85118325303 OR 2-s2.0-84867166849 OR 2-s2.0-85082504256 OR 2-s2.0-0034937048 OR 2-s2.0-84893283381 OR 2-s2.0-84866539356 OR 2-s2.0-85168003972 OR 2-s2.0-85026251268 OR 2-s2.0-85141843491 OR 2-s2.0-85063259484 OR 2-s2.0-85180104735 OR 2-s2.0-85107572409 OR 2-s2.0-85004011363 OR 2-s2.0-85012434763 OR 2-s2.0-85006164687 OR 2-s2.0-85106631393 OR 2-s2.0-85142226256 OR 2-s2.0-85071035980 OR 2-s2.0-17944386604 OR 2-s2.0-84883191694 OR 2-s2.0-85168140007 OR 2-s2.0-0028849296 OR 2-s2.0-85049775261 OR 2-s2.0-84919707604 OR 2-s2.0-84871482119 OR 2-s2.0-85126046322 OR 2-s2.0-84876289780 OR 2-s2.0-84952684290 OR 2-s2.0-85067984893 OR 2-s2.0-85160674840 OR 2-s2.0-84958971434 OR 2-s2.0-64249164660 OR 2-s2.0-84930982023 OR 2-s2.0-84861187410 OR 2-s2.0-85074923243 OR 2-s2.0-84922227587 OR 2-s2.0-0027947421 OR 2-s2.0-44649095716 OR 2-s2.0-85059374522 OR 2-s2.0-79955937971 OR 2-s2.0-85088776770 OR 2-s2.0-85030119749 OR 2-s2.0-85122457608 OR 2-s2.0-85159907765 OR 2-s2.0-84867120774 OR 2-s2.0-55949102994 OR 2-s2.0-80051795268 OR 2-s2.0-85160033148 OR 2-s2.0-84862616844 OR 2-s2.0-85138194107 OR 2-s2.0-84874328732 OR 2-s2.0-85125334062 OR 2-s2.0-84891681750 OR 2-s2.0-0028113928 OR 2-s2.0-85172279012 OR 2-s2.0-85013687613 OR 2-s2.0-85113782913 OR 2-s2.0-84856233436 OR 2-s2.0-84863395212 OR 2-s2.0-85085905565 OR 2-s2.0-85122655667 OR 2-s2.0-84907535343 OR 2-s2.0-85088940210 OR 2-s2.0-85020077381 OR 2-s2.0-85118229058 OR 2-s2.0-85178082804 OR 2-s2.0-84937419722 OR 2-s2.0-82455212983 OR 2-s2.0-85164592263 OR 2-s2.0-85080054743 OR 2-s2.0-84922216743 OR 2-s2.0-84888241918 OR 2-s2.0-0032801041 OR 2-s2.0-85091838918 OR 2-s2.0-27644480543 OR 2-s2.0-85140732450 OR 2-s2.0-85075647855 OR 2-s2.0-85013635475 OR 2-s2.0-84891722691 OR 2-s2.0-85160852495 OR 2-s2.0-84866179449 OR 2-s2.0-85176800660 OR 2-s2.0-57149096248 OR 2-s2.0-85178434193 OR 2-s2.0-0032932606 OR 2-s2.0-0029062333 OR 2-s2.0-85137931164 OR 2-s2.0-84897025367 OR 2-s2.0-84880923343 OR 2-s2.0-85002249030 OR 2-s2.0-84921885478 OR 2-s2.0-80051942883 OR 2-s2.0-85133741303 OR 2-s2.0-85156187935 ) AND ( LIMIT-TO ( SRCTYPE, “j” ) ) AND ( LIMIT-TO (PUBSTAGE, “final” ) ) AND ( LIMIT-TO ( DOCTYPE, “ar” ) ) AND ( EXCLUDE ( PUBYEAR, 2024 ) OR EXCLUDE ( PUBYEAR, 2025 ) )
9.	Sub	VGSC as Treatment in infectious diseases (62 records)	TITLE-ABS ( “ion* channel*” ) AND ( voltage AND gated AND sodium AND channel* ) AND ( “treatment*” OR “therapeutic*” ) AND ( “infectious*” AND “disease*” ) AND NOT EID ( 2-s2.0-84927597176 OR 2-s2.0-84922010899 OR 2-s2.0-84900395214 OR 2-s2.0-85107710767 OR 2-s2.0-84962866792 OR 2-s2.0-33846673828 OR 2-s2.0-33947512419 OR 2-s2.0-85020991556 OR 2-s2.0-85013188047 OR 2-s2.0-84867125510 OR 2-s2.0-84891480845 OR 2-s2.0-84879845514 OR 2-s2.0-84942895036 OR 2-s2.0-84897059401 OR 2-s2.0-30844471383 OR 2-s2.0-85007352588 OR 2-s2.0-85039985438 OR 2-s2.0-0042536473 OR 2-s2.0-85021167561 OR 2-s2.0-79960698168 OR 2-s2.0-0037762690 OR 2-s2.0-85007368482 OR 2-s2.0-79955768829 OR 2-s2.0-84936928499 OR 2-s2.0-84951574682 OR 2-s2.0-70349335964 OR 2-s2.0-34447098802 OR 2-s2.0-85059541431 OR 2-s2.0-0030729279 OR 2-s2.0-77952309581 OR 2-s2.0-84883246797 OR 2-s2.0-85056496309 OR 2-s2.0-0025318574 OR 2-s2.0-85065493621 OR 2-s2.0-85060133773 OR 2-s2.0-0035313235 OR 2-s2.0-84962920731 OR 2-s2.0-84916207732 OR 2-s2.0-85046630803 OR 2-s2.0-84913549379 OR 2-s2.0-85007289400 OR 2-s2.0-36749015585 OR 2-s2.0-85066149701 OR 2-s2.0-79955558300 OR 2-s2.0-85073657423 OR 2-s2.0-85037690174 OR 2-s2.0-85074871955 OR 2-s2.0-84858161220 OR 2-s2.0-0023892933 OR 2-s2.0-79952262290 OR 2-s2.0-84981489482 OR 2-s2.0-84916201722 OR 2-s2.0-84890879754 OR 2-s2.0-84878393994 OR 2-s2.0-0037220090 OR 2-s2.0-84899860722 OR 2-s2.0-0036062999 OR 2-s2.0-84875794865 OR 2-s2.0-84958582085 OR 2-s2.0-84958242104 OR 2-s2.0-33750529349 OR 2-s2.0-85044159990 OR 2-s2.0-80054860325 OR 2-s2.0-84963751071 OR 2-s2.0-84893288265 OR 2-s2.0-85058928631 OR 2-s2.0-0031014473 OR 2-s2.0-84882705859 OR 2-s2.0-84880597393 OR 2-s2.0-84940413152 OR 2-s2.0-84877986414 OR 2-s2.0-0031030995 OR 2-s2.0-84883592437 OR 2-s2.0-33846264865 OR 2-s2.0-0030013925 OR 2-s2.0-84863777217 OR 2-s2.0-79959542489 OR 2-s2.0-58349114016 OR 2-s2.0-33745066809 OR 2-s2.0-84868126752 OR 2-s2.0-84866179386 OR 2-s2.0-84923819161 OR 2-s2.0-0032473367 OR 2-s2.0-70350379630 OR 2-s2.0-84925068443 OR 2-s2.0-33846188141 OR 2-s2.0-85078793098 OR 2-s2.0-84867899587 OR 2-s2.0-0036711450 OR 2-s2.0-84942886554 OR 2-s2.0-84921762521 OR 2-s2.0-84907515649 OR 2-s2.0-84864851318 OR 2-s2.0-0033066629 OR 2-s2.0-85082191409 OR 2-s2.0-84953297333 OR 2-s2.0-84945961579 OR 2-s2.0-84983788995 OR 2-s2.0-84871195861 OR 2-s2.0-85029213913 OR 2-s2.0-79955781475 OR 2-s2.0-84994959932 OR 2-s2.0-0033796095 OR 2-s2.0-84954285851 OR 2-s2.0-84896257270 OR 2-s2.0-85020011420 OR 2-s2.0-85058417690 OR 2-s2.0-85026610521 OR 2-s2.0-35648965716 OR 2-s2.0-84898772762 OR 2-s2.0-0034765967 OR 2-s2.0-85039868873 OR 2-s2.0-85083345183 OR 2-s2.0-84937808968 OR 2-s2.0-85071668624 OR 2-s2.0-85053042853 OR 2-s2.0-85007502838 OR 2-s2.0-85071777987 OR 2-s2.0-80052018704 OR 2-s2.0-84975781011 OR 2-s2.0-0027970905 OR 2-s2.0-85008474476 OR 2-s2.0-84994460502 OR 2-s2.0-84987851765 OR 2-s2.0-85070461451 OR 2-s2.0-79952082416 OR 2-s2.0-34547928925 OR 2-s2.0-85023744316 OR 2-s2.0-84889071254 OR 2-s2.0-77956830773 OR 2-s2.0-85112324505 OR 2-s2.0-84866179225 OR 2-s2.0-84922469457 OR 2-s2.0-0033016358 OR 2-s2.0-85118244016 OR 2-s2.0-85066492387 OR 2-s2.0-84869435941 OR 2-s2.0-85008702040 OR 2-s2.0-85041405270 OR 2-s2.0-85009442806 OR 2-s2.0-85065876846 OR 2-s2.0-84862023506 OR 2-s2.0-84930200978 OR 2-s2.0-84925340657 OR 2-s2.0-84895197157 OR 2-s2.0-84909583123 OR 2-s2.0-84931275456 OR 2-s2.0-85065229902 OR 2-s2.0-84911422091 OR 2-s2.0-79251630714 OR 2-s2.0-85097099229 OR 2-s2.0-84930644403 OR 2-s2.0-85131739568 OR 2-s2.0-84944047911 OR 2-s2.0-84952914192 OR 2-s2.0-84947802952 OR 2-s2.0-85111296029 OR 2-s2.0-84903791068 OR 2-s2.0-84942112073 OR 2-s2.0-0032886030 OR 2-s2.0-84930628571 OR 2-s2.0-84961684980 OR 2-s2.0-85043599838 OR 2-s2.0-84961218926 OR 2-s2.0-85130511821 OR 2-s2.0-0023296279 OR 2-s2.0-85093918416 OR 2-s2.0-50649102821 OR 2-s2.0-84867481677 OR 2-s2.0-84873371685 OR 2-s2.0-84866927563 OR 2-s2.0-34249790552 OR 2-s2.0-77953806557 OR 2-s2.0-84883057159 OR 2-s2.0-85018824585 OR 2-s2.0-85018255401 OR 2-s2.0-85048961138 OR 2-s2.0-84877691608 OR 2-s2.0-84974739765 OR 2-s2.0-33645960334 OR 2-s2.0-85097670709 OR 2-s2.0-33750951949 OR 2-s2.0-18944379387 OR 2-s2.0-85152211811 OR 2-s2.0-85149382143 OR 2-s2.0-84979917789 OR 2-s2.0-85123908483 OR 2-s2.0-85141892704 OR 2-s2.0-84962624055 OR 2-s2.0-84870893423 OR 2-s2.0-84999027826 OR 2-s2.0-85041407530 OR 2-s2.0-85078195276 OR 2-s2.0-85015811495 OR 2-s2.0-67649948710 OR 2-s2.0-34548856200 OR 2-s2.0-0035022536 OR 2-s2.0-0033736137 OR 2-s2.0-85062402027 OR 2-s2.0-0035002823 OR 2-s2.0-85131727015 OR 2-s2.0-80052003680 OR 2-s2.0-85095864874 OR 2-s2.0-85138152575 OR 2-s2.0-0035132807 OR 2-s2.0-85015359649 OR 2-s2.0-85138100456 OR 2-s2.0-85125234870 OR 2-s2.0-0034186101 OR 2-s2.0-79958289955 OR 2-s2.0-85087961945 OR 2-s2.0-85013720854 OR 2-s2.0-84886811106 OR 2-s2.0-80051981571 OR 2-s2.0-84874993259 OR 2-s2.0-84896310599 OR 2-s2.0-69649093196 OR 2-s2.0-85137719332 OR 2-s2.0-79952033330 OR 2-s2.0-34247183564 OR 2-s2.0-85063796395 OR 2-s2.0-84879191741 OR 2-s2.0-84857516171 OR 2-s2.0-77953731148 OR 2-s2.0-85119920301 OR 2-s2.0-85122473760 OR 2-s2.0-80052850827 OR 2-s2.0-85118325303 OR 2-s2.0-84867166849 OR 2-s2.0-85082504256 OR 2-s2.0-0034937048 OR 2-s2.0-84893283381 OR 2-s2.0-84866539356 OR 2-s2.0-85168003972 OR 2-s2.0-85026251268 OR 2-s2.0-85141843491 OR 2-s2.0-85063259484 OR 2-s2.0-85180104735 OR 2-s2.0-85107572409 OR 2-s2.0-85004011363 OR 2-s2.0-85012434763 OR 2-s2.0-85006164687 OR 2-s2.0-85106631393 OR 2-s2.0-85142226256 OR 2-s2.0-85071035980 OR 2-s2.0-17944386604 OR 2-s2.0-84883191694 OR 2-s2.0-85168140007 OR 2-s2.0-0028849296 OR 2-s2.0-85049775261 OR 2-s2.0-84919707604 OR 2-s2.0-84871482119 OR 2-s2.0-85126046322 OR 2-s2.0-84876289780 OR 2-s2.0-84952684290 OR 2-s2.0-85067984893 OR 2-s2.0-85160674840 OR 2-s2.0-84958971434 OR 2-s2.0-64249164660 OR 2-s2.0-84930982023 OR 2-s2.0-84861187410 OR 2-s2.0-85074923243 OR 2-s2.0-84922227587 OR 2-s2.0-0027947421 OR 2-s2.0-44649095716 OR 2-s2.0-85059374522 OR 2-s2.0-79955937971 OR 2-s2.0-85088776770 OR 2-s2.0-85030119749 OR 2-s2.0-85122457608 OR 2-s2.0-85159907765 OR 2-s2.0-84867120774 OR 2-s2.0-55949102994 OR 2-s2.0-80051795268 OR 2-s2.0-85160033148 OR 2-s2.0-84862616844 OR 2-s2.0-85138194107 OR 2-s2.0-84874328732 OR 2-s2.0-85125334062 OR 2-s2.0-84891681750 OR 2-s2.0-0028113928 OR 2-s2.0-85172279012 OR 2-s2.0-85013687613 OR 2-s2.0-85113782913 OR 2-s2.0-84856233436 OR 2-s2.0-84863395212 OR 2-s2.0-85085905565 OR 2-s2.0-85122655667 OR 2-s2.0-84907535343 OR 2-s2.0-85088940210 OR 2-s2.0-85020077381 OR 2-s2.0-85118229058 OR 2-s2.0-85178082804 OR 2-s2.0-84937419722 OR 2-s2.0-82455212983 OR 2-s2.0-85164592263 OR 2-s2.0-85080054743 OR 2-s2.0-84922216743 OR 2-s2.0-84888241918 OR 2-s2.0-0032801041 OR 2-s2.0-85091838918 OR 2-s2.0-27644480543 OR 2-s2.0-85140732450 OR 2-s2.0-85075647855 OR 2-s2.0-85013635475 OR 2-s2.0-84891722691 OR 2-s2.0-85160852495 OR 2-s2.0-84866179449 OR 2-s2.0-85176800660 OR 2-s2.0-57149096248 OR 2-s2.0-85178434193 OR 2-s2.0-0032932606 OR 2-s2.0-0029062333 OR 2-s2.0-85137931164 OR 2-s2.0-84897025367 OR 2-s2.0-84880923343 OR 2-s2.0-85002249030 OR 2-s2.0-84921885478 OR 2-s2.0-80051942883 OR 2-s2.0-85133741303 OR 2-s2.0-85156187935 ) AND ( LIMIT-TO ( SRCTYPE,”j” ) ) AND ( LIMIT-TO ( PUBSTAGE,”final” ) ) AND ( LIMIT-TO ( DOCTYPE,”ar” ) ) AND ( EXCLUDE ( PUBYEAR,2024) OR EXCLUDE ( PUBYEAR,2025) )
10.	Sub	VGSC as Treatment in deficiency diseases (186 records)	TITLE-ABS ( “ion* channel*” ) AND ( voltage AND gated AND sodium AND channel* ) AND ( “treatment*” OR “therapeutic*” ) AND ( “deficiency*” AND “disease*” ) AND NOT EID ( 2-s2.0-84927597176 OR 2-s2.0-84922010899 OR 2-s2.0-84900395214 OR 2-s2.0-85107710767 OR 2-s2.0-84962866792 OR 2-s2.0-33846673828 OR 2-s2.0-33947512419 OR 2-s2.0-85020991556 OR 2-s2.0-85013188047 OR 2-s2.0-84867125510 OR 2-s2.0-84891480845 OR 2-s2.0-84879845514 OR 2-s2.0-84942895036 OR 2-s2.0-84897059401 OR 2-s2.0-30844471383 OR 2-s2.0-85007352588 OR 2-s2.0-85039985438 OR 2-s2.0-0042536473 OR 2-s2.0-85021167561 OR 2-s2.0-79960698168 OR 2-s2.0-0037762690 OR 2-s2.0-85007368482 OR 2-s2.0-79955768829 OR 2-s2.0-84936928499 OR 2-s2.0-84951574682 OR 2-s2.0-70349335964 OR 2-s2.0-34447098802 OR 2-s2.0-85059541431 OR 2-s2.0-0030729279 OR 2-s2.0-77952309581 OR 2-s2.0-84883246797 OR 2-s2.0-85056496309 OR 2-s2.0-0025318574 OR 2-s2.0-85065493621 OR 2-s2.0-85060133773 OR 2-s2.0-0035313235 OR 2-s2.0-84962920731 OR 2-s2.0-84916207732 OR 2-s2.0-85046630803 OR 2-s2.0-84913549379 OR 2-s2.0-85007289400 OR 2-s2.0-36749015585 OR 2-s2.0-85066149701 OR 2-s2.0-79955558300 OR 2-s2.0-85073657423 OR 2-s2.0-85037690174 OR 2-s2.0-85074871955 OR 2-s2.0-84858161220 OR 2-s2.0-0023892933 OR 2-s2.0-79952262290 OR 2-s2.0-84981489482 OR 2-s2.0-84916201722 OR 2-s2.0-84890879754 OR 2-s2.0-84878393994 OR 2-s2.0-0037220090 OR 2-s2.0-84899860722 OR 2-s2.0-0036062999 OR 2-s2.0-84875794865 OR 2-s2.0-84958582085 OR 2-s2.0-84958242104 OR 2-s2.0-33750529349 OR 2-s2.0-85044159990 OR 2-s2.0-80054860325 OR 2-s2.0-84963751071 OR 2-s2.0-84893288265 OR 2-s2.0-85058928631 OR 2-s2.0-0031014473 OR 2-s2.0-84882705859 OR 2-s2.0-84880597393 OR 2-s2.0-84940413152 OR 2-s2.0-84877986414 OR 2-s2.0-0031030995 OR 2-s2.0-84883592437 OR 2-s2.0-33846264865 OR 2-s2.0-0030013925 OR 2-s2.0-84863777217 OR 2-s2.0-79959542489 OR 2-s2.0-58349114016 OR 2-s2.0-33745066809 OR 2-s2.0-84868126752 OR 2-s2.0-84866179386 OR 2-s2.0-84923819161 OR 2-s2.0-0032473367 OR 2-s2.0-70350379630 OR 2-s2.0-84925068443 OR 2-s2.0-33846188141 OR 2-s2.0-85078793098 OR 2-s2.0-84867899587 OR 2-s2.0-0036711450 OR 2-s2.0-84942886554 OR 2-s2.0-84921762521 OR 2-s2.0-84907515649 OR 2-s2.0-84864851318 OR 2-s2.0-0033066629 OR 2-s2.0-85082191409 OR 2-s2.0-84953297333 OR 2-s2.0-84945961579 OR 2-s2.0-84983788995 OR 2-s2.0-84871195861 OR 2-s2.0-85029213913 OR 2-s2.0-79955781475 OR 2-s2.0-84994959932 OR 2-s2.0-0033796095 OR 2-s2.0-84954285851 OR 2-s2.0-84896257270 OR 2-s2.0-85020011420 OR 2-s2.0-85058417690 OR 2-s2.0-85026610521 OR 2-s2.0-35648965716 OR 2-s2.0-84898772762 OR 2-s2.0-0034765967 OR 2-s2.0-85039868873 OR 2-s2.0-85083345183 OR 2-s2.0-84937808968 OR 2-s2.0-85071668624 OR 2-s2.0-85053042853 OR 2-s2.0-85007502838 OR 2-s2.0-85071777987 OR 2-s2.0-80052018704 OR 2-s2.0-84975781011 OR 2-s2.0-0027970905 OR 2-s2.0-85008474476 OR 2-s2.0-84994460502 OR 2-s2.0-84987851765 OR 2-s2.0-85070461451 OR 2-s2.0-79952082416 OR 2-s2.0-34547928925 OR 2-s2.0-85023744316 OR 2-s2.0-84889071254 OR 2-s2.0-77956830773 OR 2-s2.0-85112324505 OR 2-s2.0-84866179225 OR 2-s2.0-84922469457 OR 2-s2.0-0033016358 OR 2-s2.0-85118244016 OR 2-s2.0-85066492387 OR 2-s2.0-84869435941 OR 2-s2.0-85008702040 OR 2-s2.0-85041405270 OR 2-s2.0-85009442806 OR 2-s2.0-85065876846 OR 2-s2.0-84862023506 OR 2-s2.0-84930200978 OR 2-s2.0-84925340657 OR 2-s2.0-84895197157 OR 2-s2.0-84909583123 OR 2-s2.0-84931275456 OR 2-s2.0-85065229902 OR 2-s2.0-84911422091 OR 2-s2.0-79251630714 OR 2-s2.0-85097099229 OR 2-s2.0-84930644403 OR 2-s2.0-85131739568 OR 2-s2.0-84944047911 OR 2-s2.0-84952914192 OR 2-s2.0-84947802952 OR 2-s2.0-85111296029 OR 2-s2.0-84903791068 OR 2-s2.0-84942112073 OR 2-s2.0-0032886030 OR 2-s2.0-84930628571 OR 2-s2.0-84961684980 OR 2-s2.0-85043599838 OR 2-s2.0-84961218926 OR 2-s2.0-85130511821 OR 2-s2.0-0023296279 OR 2-s2.0-85093918416 OR 2-s2.0-50649102821 OR 2-s2.0-84867481677 OR 2-s2.0-84873371685 OR 2-s2.0-84866927563 OR 2-s2.0-34249790552 OR 2-s2.0-77953806557 OR 2-s2.0-84883057159 OR 2-s2.0-85018824585 OR 2-s2.0-85018255401 OR 2-s2.0-85048961138 OR 2-s2.0-84877691608 OR 2-s2.0-84974739765 OR 2-s2.0-33645960334 OR 2-s2.0-85097670709 OR 2-s2.0-33750951949 OR 2-s2.0-18944379387 OR 2-s2.0-85152211811 OR 2-s2.0-85149382143 OR 2-s2.0-84979917789 OR 2-s2.0-85123908483 OR 2-s2.0-85141892704 OR 2-s2.0-84962624055 OR 2-s2.0-84870893423 OR 2-s2.0-84999027826 OR 2-s2.0-85041407530 OR 2-s2.0-85078195276 OR 2-s2.0-85015811495 OR 2-s2.0-67649948710 OR 2-s2.0-34548856200 OR 2-s2.0-0035022536 OR 2-s2.0-0033736137 OR 2-s2.0-85062402027 OR 2-s2.0-0035002823 OR 2-s2.0-85131727015 OR 2-s2.0-80052003680 OR 2-s2.0-85095864874 OR 2-s2.0-85138152575 OR 2-s2.0-0035132807 OR 2-s2.0-85015359649 OR 2-s2.0-85138100456 OR 2-s2.0-85125234870 OR 2-s2.0-0034186101 OR 2-s2.0-79958289955 OR 2-s2.0-85087961945 OR 2-s2.0-85013720854 OR 2-s2.0-84886811106 OR 2-s2.0-80051981571 OR 2-s2.0-84874993259 OR 2-s2.0-84896310599 OR 2-s2.0-69649093196 OR 2-s2.0-85137719332 OR 2-s2.0-79952033330 OR 2-s2.0-34247183564 OR 2-s2.0-85063796395 OR 2-s2.0-84879191741 OR 2-s2.0-84857516171 OR 2-s2.0-77953731148 OR 2-s2.0-85119920301 OR 2-s2.0-85122473760 OR 2-s2.0-80052850827 OR 2-s2.0-85118325303 OR 2-s2.0-84867166849 OR 2-s2.0-85082504256 OR 2-s2.0-0034937048 OR 2-s2.0-84893283381 OR 2-s2.0-84866539356 OR 2-s2.0-85168003972 OR 2-s2.0-85026251268 OR 2-s2.0-85141843491 OR 2-s2.0-85063259484 OR 2-s2.0-85180104735 OR 2-s2.0-85107572409 OR 2-s2.0-85004011363 OR 2-s2.0-85012434763 OR 2-s2.0-85006164687 OR 2-s2.0-85106631393 OR 2-s2.0-85142226256 OR 2-s2.0-85071035980 OR 2-s2.0-17944386604 OR 2-s2.0-84883191694 OR 2-s2.0-85168140007 OR 2-s2.0-0028849296 OR 2-s2.0-85049775261 OR 2-s2.0-84919707604 OR 2-s2.0-84871482119 OR 2-s2.0-85126046322 OR 2-s2.0-84876289780 OR 2-s2.0-84952684290 OR 2-s2.0-85067984893 OR 2-s2.0-85160674840 OR 2-s2.0-84958971434 OR 2-s2.0-64249164660 OR 2-s2.0-84930982023 OR 2-s2.0-84861187410 OR 2-s2.0-85074923243 OR 2-s2.0-84922227587 OR 2-s2.0-0027947421 OR 2-s2.0-44649095716 OR 2-s2.0-85059374522 OR 2-s2.0-79955937971 OR 2-s2.0-85088776770 OR 2-s2.0-85030119749 OR 2-s2.0-85122457608 OR 2-s2.0-85159907765 OR 2-s2.0-84867120774 OR 2-s2.0-55949102994 OR 2-s2.0-80051795268 OR 2-s2.0-85160033148 OR 2-s2.0-84862616844 OR 2-s2.0-85138194107 OR 2-s2.0-84874328732 OR 2-s2.0-85125334062 OR 2-s2.0-84891681750 OR 2-s2.0-0028113928 OR 2-s2.0-85172279012 OR 2-s2.0-85013687613 OR 2-s2.0-85113782913 OR 2-s2.0-84856233436 OR 2-s2.0-84863395212 OR 2-s2.0-85085905565 OR 2-s2.0-85122655667 OR 2-s2.0-84907535343 OR 2-s2.0-85088940210 OR 2-s2.0-85020077381 OR 2-s2.0-85118229058 OR 2-s2.0-85178082804 OR 2-s2.0-84937419722 OR 2-s2.0-82455212983 OR 2-s2.0-85164592263 OR 2-s2.0-85080054743 OR 2-s2.0-84922216743 OR 2-s2.0-84888241918 OR 2-s2.0-0032801041 OR 2-s2.0-85091838918 OR 2-s2.0-27644480543 OR 2-s2.0-85140732450 OR 2-s2.0-85075647855 OR 2-s2.0-85013635475 OR 2-s2.0-84891722691 OR 2-s2.0-85160852495 OR 2-s2.0-84866179449 OR 2-s2.0-85176800660 OR 2-s2.0-57149096248 OR 2-s2.0-85178434193 OR 2-s2.0-0032932606 OR 2-s2.0-0029062333 OR 2-s2.0-85137931164 OR 2-s2.0-84897025367 OR 2-s2.0-84880923343 OR 2-s2.0-85002249030 OR 2-s2.0-84921885478 OR 2-s2.0-80051942883 OR 2-s2.0-85133741303 OR 2-s2.0-85156187935 ) AND ( LIMIT-TO ( SRCTYPE, “j” ) ) AND ( LIMIT-TO ( PUBSTAGE, “final” ) ) AND ( LIMIT-TO ( DOCTYPE, “ar” ) ) AND ( EXCLUDE ( PUBYEAR, 2024 ) OR EXCLUDE ( PUBYEAR, 2025 ) )
11.	Sub	VGSC as Treatment in physiological diseases (1,035 records)	TITLE-ABS ( “ion* channel*” ) AND ( voltage AND gated AND sodium AND channel* ) AND ( “treatment*” OR “therapeutic*” ) AND ( “physiological*” AND “disease*” ) AND NOT EID ( 2-s2.0-84927597176 OR 2-s2.0-84922010899 OR 2-s2.0-84900395214 OR 2-s2.0-85107710767 OR 2-s2.0-84962866792 OR 2-s2.0-33846673828 OR 2-s2.0-33947512419 OR 2-s2.0-85020991556 OR 2-s2.0-85013188047 OR 2-s2.0-84867125510 OR 2-s2.0-84891480845 OR 2-s2.0-84879845514 OR 2-s2.0-84942895036 OR 2-s2.0-84897059401 OR 2-s2.0-30844471383 OR 2-s2.0-85007352588 OR 2-s2.0-85039985438 OR 2-s2.0-0042536473 OR 2-s2.0-85021167561 OR 2-s2.0-79960698168 OR 2-s2.0-0037762690 OR 2-s2.0-85007368482 OR 2-s2.0-79955768829 OR 2-s2.0-84936928499 OR 2-s2.0-84951574682 OR 2-s2.0-70349335964 OR 2-s2.0-34447098802 OR 2-s2.0-85059541431 OR 2-s2.0-0030729279 OR 2-s2.0-77952309581 OR 2-s2.0-84883246797 OR 2-s2.0-85056496309 OR 2-s2.0-0025318574 OR 2-s2.0-85065493621 OR 2-s2.0-85060133773 OR 2-s2.0-0035313235 OR 2-s2.0-84962920731 OR 2-s2.0-84916207732 OR 2-s2.0-85046630803 OR 2-s2.0-84913549379 OR 2-s2.0-85007289400 OR 2-s2.0-36749015585 OR 2-s2.0-85066149701 OR 2-s2.0-79955558300 OR 2-s2.0-85073657423 OR 2-s2.0-85037690174 OR 2-s2.0-85074871955 OR 2-s2.0-84858161220 OR 2-s2.0-0023892933 OR 2-s2.0-79952262290 OR 2-s2.0-84981489482 OR 2-s2.0-84916201722 OR 2-s2.0-84890879754 OR 2-s2.0-84878393994 OR 2-s2.0-0037220090 OR 2-s2.0-84899860722 OR 2-s2.0-0036062999 OR 2-s2.0-84875794865 OR 2-s2.0-84958582085 OR 2-s2.0-84958242104 OR 2-s2.0-33750529349 OR 2-s2.0-85044159990 OR 2-s2.0-80054860325 OR 2-s2.0-84963751071 OR 2-s2.0-84893288265 OR 2-s2.0-85058928631 OR 2-s2.0-0031014473 OR 2-s2.0-84882705859 OR 2-s2.0-84880597393 OR 2-s2.0-84940413152 OR 2-s2.0-84877986414 OR 2-s2.0-0031030995 OR 2-s2.0-84883592437 OR 2-s2.0-33846264865 OR 2-s2.0-0030013925 OR 2-s2.0-84863777217 OR 2-s2.0-79959542489 OR 2-s2.0-58349114016 OR 2-s2.0-33745066809 OR 2-s2.0-84868126752 OR 2-s2.0-84866179386 OR 2-s2.0-84923819161 OR 2-s2.0-0032473367 OR 2-s2.0-70350379630 OR 2-s2.0-84925068443 OR 2-s2.0-33846188141 OR 2-s2.0-85078793098 OR 2-s2.0-84867899587 OR 2-s2.0-0036711450 OR 2-s2.0-84942886554 OR 2-s2.0-84921762521 OR 2-s2.0-84907515649 OR 2-s2.0-84864851318 OR 2-s2.0-0033066629 OR 2-s2.0-85082191409 OR 2-s2.0-84953297333 OR 2-s2.0-84945961579 OR 2-s2.0-84983788995 OR 2-s2.0-84871195861 OR 2-s2.0-85029213913 OR 2-s2.0-79955781475 OR 2-s2.0-84994959932 OR 2-s2.0-0033796095 OR 2-s2.0-84954285851 OR 2-s2.0-84896257270 OR 2-s2.0-85020011420 OR 2-s2.0-85058417690 OR 2-s2.0-85026610521 OR 2-s2.0-35648965716 OR 2-s2.0-84898772762 OR 2-s2.0-0034765967 OR 2-s2.0-85039868873 OR 2-s2.0-85083345183 OR 2-s2.0-84937808968 OR 2-s2.0-85071668624 OR 2-s2.0-85053042853 OR 2-s2.0-85007502838 OR 2-s2.0-85071777987 OR 2-s2.0-80052018704 OR 2-s2.0-84975781011 OR 2-s2.0-0027970905 OR 2-s2.0-85008474476 OR 2-s2.0-84994460502 OR 2-s2.0-84987851765 OR 2-s2.0-85070461451 OR 2-s2.0-79952082416 OR 2-s2.0-34547928925 OR 2-s2.0-85023744316 OR 2-s2.0-84889071254 OR 2-s2.0-77956830773 OR 2-s2.0-85112324505 OR 2-s2.0-84866179225 OR 2-s2.0-84922469457 OR 2-s2.0-0033016358 OR 2-s2.0-85118244016 OR 2-s2.0-85066492387 OR 2-s2.0-84869435941 OR 2-s2.0-85008702040 OR 2-s2.0-85041405270 OR 2-s2.0-85009442806 OR 2-s2.0-85065876846 OR 2-s2.0-84862023506 OR 2-s2.0-84930200978 OR 2-s2.0-84925340657 OR 2-s2.0-84895197157 OR 2-s2.0-84909583123 OR 2-s2.0-84931275456 OR 2-s2.0-85065229902 OR 2-s2.0-84911422091 OR 2-s2.0-79251630714 OR 2-s2.0-85097099229 OR 2-s2.0-84930644403 OR 2-s2.0-85131739568 OR 2-s2.0-84944047911 OR 2-s2.0-84952914192 OR 2-s2.0-84947802952 OR 2-s2.0-85111296029 OR 2-s2.0-84903791068 OR 2-s2.0-84942112073 OR 2-s2.0-0032886030 OR 2-s2.0-84930628571 OR 2-s2.0-84961684980 OR 2-s2.0-85043599838 OR 2-s2.0-84961218926 OR 2-s2.0-85130511821 OR 2-s2.0-0023296279 OR 2-s2.0-85093918416 OR 2-s2.0-50649102821 OR 2-s2.0-84867481677 OR 2-s2.0-84873371685 OR 2-s2.0-84866927563 OR 2-s2.0-34249790552 OR 2-s2.0-77953806557 OR 2-s2.0-84883057159 OR 2-s2.0-85018824585 OR 2-s2.0-85018255401 OR 2-s2.0-85048961138 OR 2-s2.0-84877691608 OR 2-s2.0-84974739765 OR 2-s2.0-33645960334 OR 2-s2.0-85097670709 OR 2-s2.0-33750951949 OR 2-s2.0-18944379387 OR 2-s2.0-85152211811 OR 2-s2.0-85149382143 OR 2-s2.0-84979917789 OR 2-s2.0-85123908483 OR 2-s2.0-85141892704 OR 2-s2.0-84962624055 OR 2-s2.0-84870893423 OR 2-s2.0-84999027826 OR 2-s2.0-85041407530 OR 2-s2.0-85078195276 OR 2-s2.0-85015811495 OR 2-s2.0-67649948710 OR 2-s2.0-34548856200 OR 2-s2.0-0035022536 OR 2-s2.0-0033736137 OR 2-s2.0-85062402027 OR 2-s2.0-0035002823 OR 2-s2.0-85131727015 OR 2-s2.0-80052003680 OR 2-s2.0-85095864874 OR 2-s2.0-85138152575 OR 2-s2.0-0035132807 OR 2-s2.0-85015359649 OR 2-s2.0-85138100456 OR 2-s2.0-85125234870 OR 2-s2.0-0034186101 OR 2-s2.0-79958289955 OR 2-s2.0-85087961945 OR 2-s2.0-85013720854 OR 2-s2.0-84886811106 OR 2-s2.0-80051981571 OR 2-s2.0-84874993259 OR 2-s2.0-84896310599 OR 2-s2.0-69649093196 OR 2-s2.0-85137719332 OR 2-s2.0-79952033330 OR 2-s2.0-34247183564 OR 2-s2.0-85063796395 OR 2-s2.0-84879191741 OR 2-s2.0-84857516171 OR 2-s2.0-77953731148 OR 2-s2.0-85119920301 OR 2-s2.0-85122473760 OR 2-s2.0-80052850827 OR 2-s2.0-85118325303 OR 2-s2.0-84867166849 OR 2-s2.0-85082504256 OR 2-s2.0-0034937048 OR 2-s2.0-84893283381 OR 2-s2.0-84866539356 OR 2-s2.0-85168003972 OR 2-s2.0-85026251268 OR 2-s2.0-85141843491 OR 2-s2.0-85063259484 OR 2-s2.0-85180104735 OR 2-s2.0-85107572409 OR 2-s2.0-85004011363 OR 2-s2.0-85012434763 OR 2-s2.0-85006164687 OR 2-s2.0-85106631393 OR 2-s2.0-85142226256 OR 2-s2.0-85071035980 OR 2-s2.0-17944386604 OR 2-s2.0-84883191694 OR 2-s2.0-85168140007 OR 2-s2.0-0028849296 OR 2-s2.0-85049775261 OR 2-s2.0-84919707604 OR 2-s2.0-84871482119 OR 2-s2.0-85126046322 OR 2-s2.0-84876289780 OR 2-s2.0-84952684290 OR 2-s2.0-85067984893 OR 2-s2.0-85160674840 OR 2-s2.0-84958971434 OR 2-s2.0-64249164660 OR 2-s2.0-84930982023 OR 2-s2.0-84861187410 OR 2-s2.0-85074923243 OR 2-s2.0-84922227587 OR 2-s2.0-0027947421 OR 2-s2.0-44649095716 OR 2-s2.0-85059374522 OR 2-s2.0-79955937971 OR 2-s2.0-85088776770 OR 2-s2.0-85030119749 OR 2-s2.0-85122457608 OR 2-s2.0-85159907765 OR 2-s2.0-84867120774 OR 2-s2.0-55949102994 OR 2-s2.0-80051795268 OR 2-s2.0-85160033148 OR 2-s2.0-84862616844 OR 2-s2.0-85138194107 OR 2-s2.0-84874328732 OR 2-s2.0-85125334062 OR 2-s2.0-84891681750 OR 2-s2.0-0028113928 OR 2-s2.0-85172279012 OR 2-s2.0-85013687613 OR 2-s2.0-85113782913 OR 2-s2.0-84856233436 OR 2-s2.0-84863395212 OR 2-s2.0-85085905565 OR 2-s2.0-85122655667 OR 2-s2.0-84907535343 OR 2-s2.0-85088940210 OR 2-s2.0-85020077381 OR 2-s2.0-85118229058 OR 2-s2.0-85178082804 OR 2-s2.0-84937419722 OR 2-s2.0-82455212983 OR 2-s2.0-85164592263 OR 2-s2.0-85080054743 OR 2-s2.0-84922216743 OR 2-s2.0-84888241918 OR 2-s2.0-0032801041 OR 2-s2.0-85091838918 OR 2-s2.0-27644480543 OR 2-s2.0-85140732450 OR 2-s2.0-85075647855 OR 2-s2.0-85013635475 OR 2-s2.0-84891722691 OR 2-s2.0-85160852495 OR 2-s2.0-84866179449 OR 2-s2.0-85176800660 OR 2-s2.0-57149096248 OR 2-s2.0-85178434193 OR 2-s2.0-0032932606 OR 2-s2.0-0029062333 OR 2-s2.0-85137931164 OR 2-s2.0-84897025367 OR 2-s2.0-84880923343 OR 2-s2.0-85002249030 OR 2-s2.0-84921885478 OR 2-s2.0-80051942883 OR 2-s2.0-85133741303 OR 2-s2.0-85156187935 ) AND ( LIMIT-TO ( SRCTYPE, “j” ) ) AND ( LIMIT-TO ( PUBSTAGE, “final” ) ) AND ( LIMIT-TO ( DOCTYPE, “ar” ) ) AND ( EXCLUDE ( PUBYEAR, 2024 ) OR EXCLUDE ( PUBYEAR, 2025 ) )

## Figures and Tables

**Table 1:** The top 10 most productive journal

Rank	Journal	TP (%)	Cite Score	The most cited article	Times cited	FWCI	Publisher
1	*PLOS ONE*	84 (2.64)	6.2 (2023)	RNA-Seq analysis of human trigeminal and dorsal root ganglia with a focus on chemoreceptors	140	2.42	Public Library of Science
2	*Journal of Biological Chemistry*	68 (2.15)	8.5 (2023)	Purification and characterization of a unique, potent, peptidyl probe for the high conductance calcium-activated potassium channel from venom of the scorpion Buthus tamulus	647	N/A	American Society for Biochemistry and Molecular Biology Inc.
3	*Journal of Neuroscience*	66 (2.08)	9.3 (2023)	Selective antagonism of GluR5 kainate-receptor-mediated synaptic currents by topiramate in rat basolateral amygdala neurons	196	2.82	Society for Neuroscience
4	*Proceedings of the National Academy of Sciences of The United States of America*	66 (2.08)	19 (2023)	Identification of gene expression profile of dorsal root ganglion in the rat peripheral axotomy model of neuropathic pain	461	2.42	National Academy of Sciences
5	*Journal of Physiology*	62 (1.95)	9.7 (2023)	Electrophysiological properties of human mesenchymal stem cells	193	3.59	John Wiley and Sons
6	*British Journal of Pharmacology*	59 (1.86)	15.4 (2023)	The concise guide to pharmacology 2019/20: introduction and other protein targets	371	28.64	John Wiley and Sons
7	*Scientific Reports*	47 (1.48)	7.5 (2023)	Ultrasound modulates ion channel currents	235	3.99	Springer Nature
8	*Journal of Neurophysiology*	39 (1.23)	4.8 (2023)	Simulated seizures and spreading depression in a neuron model incorporating interstitial space and ion concentrations	229	1.02	American Physiological Society
9	*Brain Research*	38 (1.19)	5.9 (2023)	Studies on the mechanism of action of the novel anticonvulsant lamotrigine (Lamictal) using primary neuroglial cultures from rat cortex	238	N/A	Elsevier
10	*Neuroscience*	38 (1.19)	6.2 (2023)	Seizures and neurodegeneration induced by 4-aminopyridine in rat hippocampus in vivo: Role of glutamate- and GABA-mediated neurotransmission and of ion channels	171	1.24	Elsevier

**Table 2:** The top 15 most productive countries and academic institutions in publications on VGSC as an ion channel in therapeutics

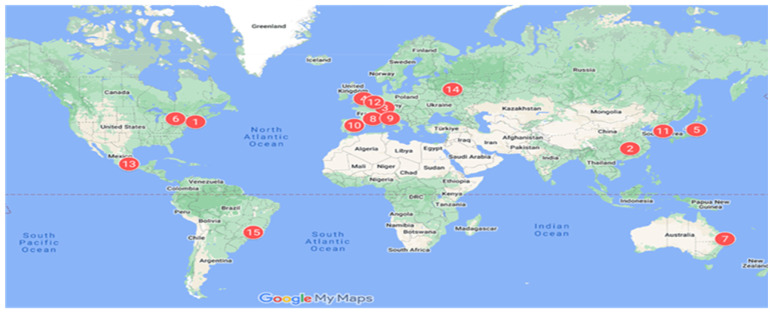					

Rank	Country	TPc	SCP (%)	The most productive academic institution	TPi
1	USA	1,318	64.64	Harvard Medical School	32
2	China	452	69.24	Hunan Normal University	21
3	Germany	330	44.84	Eberhard Karls Universität Tübingen	14
4	UK	292	37.67	University of Cambridge	12
5	Japan	195	69.74	Tohoku University	9
6	Canada	173	42.19	University of Toronto	10
7	Australia	162	45.68	The University of Queensland	29
8	France	157	43.31	Université de Montpellier	13
9	Italy	119	39.50	Università degli Studi di Firenze	8
10	Spain	73	34.25	Universidad Autónoma de Madrid	4
11	South Korea	71	71.83	Seoul National University College of Medicine	9
12	Belgium	69	5.80	KU Leuven	2
13	Mexico	61	57.38	Universidad Nacional Autónoma de México	14
14	Russia	56	53.57	Lomonosov Moscow State University	6
15	Brazil	55	36.36	Universidade Federal de São Paulo	5

TPc = total publication of a country; SCP = single-country publication; TPi = total publication of an academic institution

**Table 3:** List of the top 10 most prolific author

Rank	Author	Scopus author ID	Year of 1st publication*	Total publication	*h*-index	Total citation	Current affiliation	Country
1	Jan Tytgat	7005089896	1988^a^	37	52	515	KU Leuven	Belgium
2	Steve Peigneur	24559357200	2008^b^	34	32	410	KU Leuven	Belgium
3	Glenn F. King	7402995101	1983^a^	24	69	964	The University of Queensland	Australia
4	Richard James Lewis	55457251800	1981^b^	24	74	431	The University of Queensland	Australia
5	Sulayman Dib-Hajj	7006690341	1992^b^	16	82	913	Yale School of Medicine	USA
6	Irina Vetter	14020709200	2006^a^	16	47	302	The University of Queensland	Australia
7	Michael John Ackerman	7102657891	1991^b^	15	135	1,610	Mayo Clinic College of Medicine and Science	USA
8	Michael Levin	8880226800	1994^a^	15	77	689	Tufts University	USA
9	Paul F Alewood	7004527669	1973^a^	13	71	374	The University of Queensland	Australia
10	Guirong Li	8224611300	1983^a^	13	40	575	Xiamen University	China

* Role in co-authorship;

a First author;

b Co-author
